# Prevalence, resistance pattern, and molecular characterization of *Staphylococcus aureus* isolates from healthy animals and sick populations in Henan Province, China

**DOI:** 10.1186/s13099-018-0254-9

**Published:** 2018-07-17

**Authors:** Baoguang Liu, Huarun Sun, Yushan Pan, Yajun Zhai, Tian Cai, Xiaoling Yuan, Yanling Gao, Dandan He, Jianhua Liu, Li Yuan, Gongzheng Hu

**Affiliations:** 1 College of Animal Husbandry and Veterinary Science, Henan Agricultural University, No. 95 Wenhua Road, Zhengzhou, China; 2 Neonatal Intensive Care Unit, First Affiliated Hospital of Zhengzhou University, No. 1 Jianshe Road, Zhengzhou, China; 3 Animal Husbandry Bureau of Henan Province, No. 91 Jingsan Road, Zhengzhou, China

**Keywords:** *Staphylococcus aureus*, MRSA, Antimicrobial resistance, PFGE, *Spa* typing

## Abstract

**Background:**

*Staphylococcus aureus* is one of the most prevalent pathogens and a causative agent of a variety of infections in humans and animals. A total of 640 samples were collected from healthy animals and patients from 2013 to 2014 in Henan Province, China, to investigate the prevalence and perform molecular characterization of *S. aureus*. Antimicrobial resistance and virulence genes were determined and pulsed-field gel electrophoresis (PFGE) and *staphylococcal* cassette chromosome *mec* (SCC*mec*) typing were performed.

**Results:**

Overall, 22.3% (n = 143) of the samples were positive for *S. aureus*. The prevalence of methicillin-resistant *S. aureus* (MRSA) was 5.59%. Capsular polysaccharide locus type 5 (*Cap*5; 56.64%) was the dominant serotype. *S. aureus* strains showed high resistance to penicillin (96.50%), ciprofloxacin (52.45%), amikacin (67.83%), erythromycin (96.50%), lincomycin (97.20%), and tetracycline (68.53%) and 109 (76.2%) isolates harbored six or more tested resistance genes. The most predominant resistance genes were *aphA* (52.45%), *ermC* (53.15%), and *tetM* (52.45%). Eighty-seven (60.8%) isolates harbored six or more tested virulence genes. The most predominant enterotoxin genes were *sed* (20.28%), *sej* (20.98%), *sep* (14.69%), and *set* (37.76%). The prevalence of *lukED* gene was (57.34%), and a small number of isolates carried *pvl* (5.59%) and *TSST*-*1* (2.80%). A total of 130 (82.52%) isolates could be typed by PFGE with *SmaI* digestion. PFGE demonstrated that 45 different patterns (P) that were grouped into 17 pulsotypes and 28 separate pulsotypes using a 90% cut-off value. A total of 118 (82.52%) isolates were successfully typed by *spa*, and 26 *spa* types were identified, t15075 (14.00%) and t189 (12.59%) were the most common types. SCC*mec* types were detected from eight MRSA isolates, with the most prevalent type being SCC*mec* IVa. MRSA-SCC*mec* Iva-*t437* was observed in human isolates.

**Conclusion:**

This study revealed a high prevalence of *S. aureus* in healthy animals and patients from Henan Province, China. Resistant *S. aureus* exhibited varying degrees of multidrug resistance. The presence of antibiotic resistance and virulence genes may facilitate the spread of *S. aureus* strains and pose a potential threat to public health, highlighting the need for vigilant monitoring of these isolates at the human–animal interface.

**Electronic supplementary material:**

The online version of this article (10.1186/s13099-018-0254-9) contains supplementary material, which is available to authorized users.

## Background

*Staphylococcus aureus*, a Gram-positive bacterium, is a causative agent of a variety of infections in humans and animals [1]. Many of the illnesses of humans, such as pneumonia and endocarditis, are related to *S. aureus* [2]. In animals, *S. aureus* is associated with bovine mastitis, one of the most cost-intensive diseases in the food industry. Bovine mastitis is an infectious disease responsible for significant financial losses to dairy and food farmers worldwide [3]. Since methicillin-resistant *S. aureus* (MRSA) was first reported in the United Kingdom, it has become a particular public threat to human health, and various hospital-associated MRSA (HA-MRSA) clones have been disseminated worldwide [4]. Since the 1990s, community-associated MRSA (CA-MRSA) has emerged as a serious health problem worldwide [5], first in communities and later in healthcare facilities. However, livestock-associated MRSA (LA-MRSA) clones,such as LA-MRSA ST398 may be transmitted to humans and have posed public health concerns [6]. Therefore, it is imperative to perform surveillance at the interface between human and animal hosts to explore human health risks [7].

The emergence of multidrug resistant (MDR) strains poses several challenges to the clinical facilities [8]. In particular, the increasing prevalence of antimicrobial-resistant *S. aureus* serves as a threat to the healthcare system [9, 10]. On the other hand, most *S. aureus* strains are able to produce a large number of virulence factors, including *staphylococcal* enterotoxins (SEs), exfoliative toxin (ET) and toxic shock syndrome toxin-1 (*TSST*-*1*) genes. Moreover, the production of SEs is particularly significant, as the ingestion of the preformed toxins is a major cause of foodborne poisoning wordwide [11]. *S. aureus* has not only has been isolated from raw milk, a potential reservoir of *S. aureus*, but also from the environments and workers of dairy farms [12, 13]. In addition, studies have reported that some *S. aureus* strains persist in powdered infant formula [1]. However, reports on *S. aureus* isolation from raw milk of healthy animals are relatively scanty. Very little is known about the antimicrobial susceptibility, resistance genes of enterotoxigenic *S. aureus* strains, and the prevalence and molecular characterization of *S. aureus* isolates.

The aim of the present study was to investigate the prevalence and perform molecular characterization of *S. aureus* isolates from healthy animals and patients in Henan Province, China. Furthermore, we evaluated the antimicrobial susceptibility, resistance genes, virulence genes of these isolates and characterize the molecular types by pulsed-field gel electrophoresis (PFGE), *spa*, and SCC*mec*.

## Results

### Isolation and identification of *S. aureus*

A total of 143 (22.3%, 95% confidence interval [CI] 19.1–25.6) *S. aureus* isolates were recovered from 640 samples in Henan province between 2013 and 2014 (Table 1). Two types of samples including animal samples (n = 548, 130 isolates, 23.7%, 95% CI 20.1–27.3) and patient samples (n = 92, 13 isolates, 14.1%, 95% CI 6.9–21.4) were obtained. A total of 548 animal samples included 350 (22.9%, 95% CI 18.4–27.3) raw milk samples, 86 (25.6%, 95% CI 16.2–35.0) swine samples, 70 (21.4%, 95% CI 11.6–31.3) chicken samples and 42 (31.0%, 95% CI 16.4–45.5) duck samples. Of 92 patient samples, 48 (27.1%, 95% CI 14.0–40.1) were obtained from the First Affiliated Hospital of Zhengzhou University and 44 (0.0%, 95% CI 0.0–0.0), from the Henan Province People’s Hospital. Table 1 shows the prevalence of *S. aureus* in animals (23.7%), with a higher rate in chicken and duck (p > 0.05); in patients (14.1%), with differences rate in chicken and pork (p < 0.05).

**Table 1:** Prevalence and detection of *nuc* and *mecA* of *S. aureus* isolates from animals and hospitals

Source	Sampling sites	No. of samples	Prevalence (%)	95% Cl	Specific gene (%)	
					*nuc*	*mecA*
Animal (healthy)						
Cow (raw milk)	8	350	22.9 (80/350)	18.4–27.3	22.9 (80/350)	1.25 (1/80)
Swine (nasal swab)	2	86	25.6 (22/86)	16.2–35.0	25.6 (22/86)	0 (0/22)
Chicken (faecal swab)	2	70	21.4 (15/70)	11.6–31.3	21.4 (15/70)	6.67 (1/15)
Duck (faecal swab)	2	42	31.0 (13/42)	16.4–45.5	31.0 (13/42)	0 (0/13)
Total	13	548	23.7 (130/548)	20.1–27.3	23.7 (130/548)	1.54 (2/130)
Human (patients)						
A^a^ (specimens)	1	48	27.1 (13/48)	14.0–40.1	27.1 (13/48)	46.16 (6/13)
B^b^ (specimens)	1	44	0 (0/44)	0.0–0.0	0 (0/44)	0 (0/0)
Total	2	92	14.1 (13/92)	6.9–21.4	14.1 (13/92)	46.16 (6/13)
All total	15	640	22.3 (143/640)	19.1–25.6	22.3 (143/640)	5.59 (8/143)

^a^ The First Affiliated Hospital of Zhengzhou University

^b^ Henan Province People’s Hospital

### Prevalence of MRSA

As shown in Table 1, the prevalence of MRSA was 5.59% (8/143). Eight strains carrying *mecA* were classified as MRSA. The sources of these strains were as follows: 1 strain from raw milk, 1 from chicken, and 6 from patients. The prevalence of raw milk isolates, chicken isolates, and patient isolates were 1.25% (1/80), 6.67% (1/15), and 46.15% (6/13), respectively. Of note, the prevalence of patient isolates was significantly higher than that of raw milk isolates and chicken isolates.

### Determination of *S. aureus* serotypes

Eighty-one strains (56.64%, 81/143) were capsular polysaccharide locus type 5 (*Cap*5), 36 strains (25.17%, 36/143) were identified as *Cap*8 type, and the remaining 26 strains (18.18%, 26/143) were *Cap* non-typeable. Therefore, *Cap*5 was the dominant serotype observed.

### Antimicrobial susceptibility testing

The results of the antimicrobial susceptibility of all *S. aureus* strains are listed in Table 2. Most *S. aureus* strains showed high resistance to penicillin (138, 96.50%), ciprofloxacin (75, 52.45%), enrofloxacin (81, 56.64%), gentamicin (116, 81.12%), amikacin (97, 67.83%), tylosin (109, 76.22%), erythromycin (138, 96.50%), lincomycin (139, 97.20%), tetracycline (98, 68.53%), and olaquindox (137, 95.80%). Moderate resistance was observed for florfenicol, doxycyclin, mequindox, rifampicin and bacitracin, as evident from a resistance of 28.67, 46.15, 18.18, 51.05, and 24.48%, respectively. The rate of resistance to fosfomycin and linezolid was below 20%. None of the eight *mecA*-positive isolates was susceptible to oxacillin. Some *S. aureus* isolates were deemed methicillin-resistant by susceptibility testing but lacked *mecA* gene. All *S. aureus* isolates were susceptible to vancomycin. In addition, tigecycline, a new class of glycylcyclines, showed good activity against *S. aureus* with a minimum inhibitory concentration (MIC_50_) of 0.5 μg/mL. Tigecycline is known to exhibit broad-spectrum activity against most Gram-positive bacteria.

**Table 2:** Distribution of MICs and susceptibility of *S. aureus* isolates (*n *= 143)

Antimi crobial	Distribution of MICs (μg/mL)													
	512^a^	256^a^	128^a^	64	32	16	8	4	2	1	0.5	0.25^b^	0.125^b^	0.0625^b^
PEN		26	8	16	20	15	12	13	9	5	5	9 ‖	5 |	
MEC	8	16	17	15	12	6 ‖	36 |	12	2	18	1			
OXA	30	17	17	15	17	13	9	10 ‖	6 |	1	5	3		
CRO			38	16 ‖	22	19	14 |	8	13	12	1			
CEF			65	14	11	21	9 ‖	4	4 |	7	7	1		
CEQ			20	21	15	10	29 ‖	15	10 |	13	7	3		
CEP			54	32	23 ‖	7	8 |	6	11	2				
IMP			13	7	15	27	29	12 ‖	9	10 |	3	4	5	9
X			28	18	32	24 ‖	15	17 |	4	5				
Y			9	1	17 ‖	33	27	27 |	18	9	1	1		
CIP			4	6	25	15	14	11 ‖	9	25 |	19	14	1	
ENO			4	10	22	12	12	21 ‖	13	19	18 |	9	3	
LEV			1	16	20	14	18	14 ‖	20	24 |	5	8	3	
GEN	56	8	5	7	13	27 ‖	10	5 |	6	5	1			
AMK			63	34 ‖	16	7 |	11	7	3	2				
NEM			79	14	20	6 ‖	4	5 |	5	4	3	2	1	
CHL			142		‖		1 |							
FFC			18	17	6 ‖	3	30	59	9 |	1				
TYL			105	4	‖	4	|	4	9	13	3	1		
ERY			133	1	1	1	2 ‖	1		1	|	2		1
LIN			116	3	9	8	3	‖		3	1 |			
TET		3	24	44	16	11 ‖	8	13 |	8	11	5			
TER			59	30	10	6 ‖	16	3 |	5	7	6	1		
DOX				7	25	34 ‖	26	15 |	14	6	3	5	7	1
TGC^d^							3	14	17	21 ‖	21 |	36	21	10
OLA^e^			112	25‖	4	2 |								
MEQ^e^			1	25‖	63	42 |	9	3						
FOS	10	11 ‖	10	22 |	54	18	9	4	2	2	1			
RIF			14	16	24	4	7	8 ‖	19	20 |	12	5	5	9
VAN					‖	1	19	40 |	24	41	22	18	5	9
BAT^f^	20	15 ‖	35	13	1	27	24	4	3	1				
APT			135	8										
LZD				2	9	4	8 ‖	8 |	34	35	29	8	2	4

Antimi crobial	MIC range (μg/mL)	Resistance breakpoints^c^ (μg/mL)	Sensitivity breakpoints (μg/mL)	*S. aureus* (*n *= 143)				95% Cl
				R (%)	S (%)	MIC_50_ (μg/mL)	MIC_90_ (μg/mL)	
PEN	0.125–> 256	≥ 0.25	≤ 0.12	96.50	3.50	16	256	0.4–6.5
MEC	0.5–> 256	≥ 16	≤ 8	51.75	48.25	16	256	40.0–56.5
OXA	0.25–> 512	≥ 4	≤ 2	89.51	10.49	64	512	5.4–15.6
CRO	0.5–> 128	≥ 64	≤ 8	37.76	62.24	32	128	25.7–41.4
CEF	0.25–> 128	≥ 8	≤ 2	83.92	13.29	64	128	7.7–18.9
CEQ	0.25–> 128	≥ 8	≤ 2	66.43	23.08	8	128	16.1–30.1
CEP	1–> 128	≥ 32	≤ 8	76.22	18.88	64	128	12.4–25.4
IMP	< 0.063–> 128	≥ 4	≤ 1	72.73	21.68	8	64	14.8–28.5
X	1–> 128	≥ 16	≤ 4	71.33	18.18	32	128	11.8–24.6
Y	0.25–> 128	≥ 32	≤ 4	18.88	39.16	8	32	31.1–47.3
CIP	0.125–> 128	≥ 4	≤ 1	52.45	41.26	4	32	33.1–49.4
ENO	0.125–128	≥ 4	≤ 0.5	56.64	20.98	4	32	14.2–27.7
LEV	0.125–> 128	≥ 4	≤ 1	58.04	27.97	4	64	20.5–35.4
GEN	0.5–> 512	≥ 16	≤ 4	81.12	11.89	64	512	6.5–17.3
AMK	1–> 128	≥ 64	≤ 16	67.83	20.98	64	128	14.2–27.7
NEM	0.125–> 128	≥ 16	≤ 4	83.22	13.99	128	128	8.2–19.7
CHL	8–> 128	≥ 32	≤ 8	99.30	0.70	128	128	0.7–2.1
FFC	1–> 128	≥ 32	≤ 2	28.67	6.99	8	128	2.8–11.2
TYL	0.25–> 128	≥ 32	≤ 8	76.22	20.98	128	128	14.2–27.7
ERY	< 0.063–> 128	≥ 8	≤ 0.5	96.50	2.10	128	128	0.3–4.5
LIN	0.5–> 128	≥ 4	≤ 0.5	97.20	0.70	128	128	0.7–2.1
TET	0.5–256	≥ 16	≤ 4	68.53	25.87	32	128	18.6–33.1
TER	0.5–> 128	≥ 16	≤ 4	73.43	15.38	64	128	9.4–21.4
DOX	< 0.063–64	≥ 16	≤ 4	46.15	35.66	8	32	27.7–43.6
TGC^d^	< 0.063–8	>0.5	≤ 0.5	38.46	61.54	0.5	4	53.5–69.6
OLA^e^	16–> 128	≥ 64	≤ 16	95.80	1.40	128	128	0.5–3.3
MEQ^e^	2–128	≥ 64	≤ 16	18.18	37.76	32	64	29.7–45.8
FOS	0.5–> 512	≥ 256	≤ 64	14.69	78.32	32	256	71.5–85.2
RIF	< 0.063–> 128	≥ 4	≤ 1	51.05	35.66	4	64	27.7–43.6
VAN	< 0.063–16	≥ 32	≤ 4	0	86.01	1	8	80.3–91.8
BAT^f^	1–> 512	≥ 256	–	24.48	–	64	512	–
APT	64–> 128	–	–	–	–	128	128	–
LZD	< 0.063–64	≥ 8	≤ 4	16.08	83.92	1	16	77.8–90.0

*PEN* penicillin, *MEC* methicillin, *OXA* oxacillin, *CRO* ceftriaxone, *CEF* ceftiofur, *CEQ* cefquinome, *CEP* cefepime, *IMP* imipenem, *X* cefoperazone and sulbactam sodium (2:1), *Y* piperacillin and tazobactam sodium (4:1), *CIP* ciprofloxacin, *ENO* enrofloxacin, *LEV* levofloxacin, *GEN* gentamicin, *AMK* amikacin, *NEM* neomycin, *CHL* chloramphenicol, *FFC* florfenicol, *TYL* tylosin, *ERY* erythromycin, *LIN* lincomycin, *TET* tetracycline, *TER* terramycine, *DOX* doxycyclin, *TGC* tigecycline, *OLA* olaquindox, *MEQ* mequindox, *FOS* fosfomycin, *RIF* rifampicin, *VAN* vancomycin, *BAT* Bacitracin, *APT* antimicrobial peptide, *LZD* linezolid

^a^Including higher than this tested MIC value

^b^Including lower than this tested MIC value

^c^MIC (µg/mL) results were calculated according to CLSI (2013) breakpoint criteria

^d^The breakpoints for tigecycline was interpreted according to criteria of the European Committee on Antimicrobial Susceptibility Testing (EUCAST)

^e^The breakpoints for olaquindox and mequindox were based on Reference values [56]

^f^Reference the reports [39]

Multidrug resistance was defined as resistance to three or more different classes of antimicrobials. A total of 143 *S. aureus* strains exhibited varying degrees of Multidrug resistance (Table 3). In total, 4.20, 3.50, and 6.29% of strains were resistant to three (p > 0.05), four (p < 0.01), and five (p > 0.05) drug classes, respectively. However, 11.89% of strains were resistant to six (p > 0.05) and seven (p > 0.05) drug classes. In addition, 23.08, 13.29, 18.18, and 7.69% of strains were resistant to eight (p < 0.05), nine (p > 0.05), 10 (p < 0.01), and 11 (p < 0.01) drug classes, respectively.

**Table 3:** Distribution of multidrug-resistance in *S. aureus* isolates (*n *= 143)

Resistance pattern	Animal isolates				Human isolates (n = 13)	Total (%)	*p*-value^a^
	Cow (n = 80)	Swine (n = 22)	Chicken (n = 15)	Duck (n = 13)			
**三**	4	1			1	6 (4.20)	0.78
四	2		3			5 (3.50)	0.007
五	6		1		2	9 (6.29)	0.358
六	12	2	1	1	1	17 (11.89)	0.785
七	9	5		1	2	17 (11.89)	0.298
八	25	6	1		1	33 (23.08)	0.025
九	11	4	1	1	2	19 (13.29)	0.836
十	9	3	7	5	2	26 (18.18)	0.005
十一	2	1	1	5	2	11 (7.69)	0.000

^a^The positive rates of resistance pattern among human isolates were compared with those among non-human isolates

### Prevalence of resistance genes and integrons

**Fig. 1:**
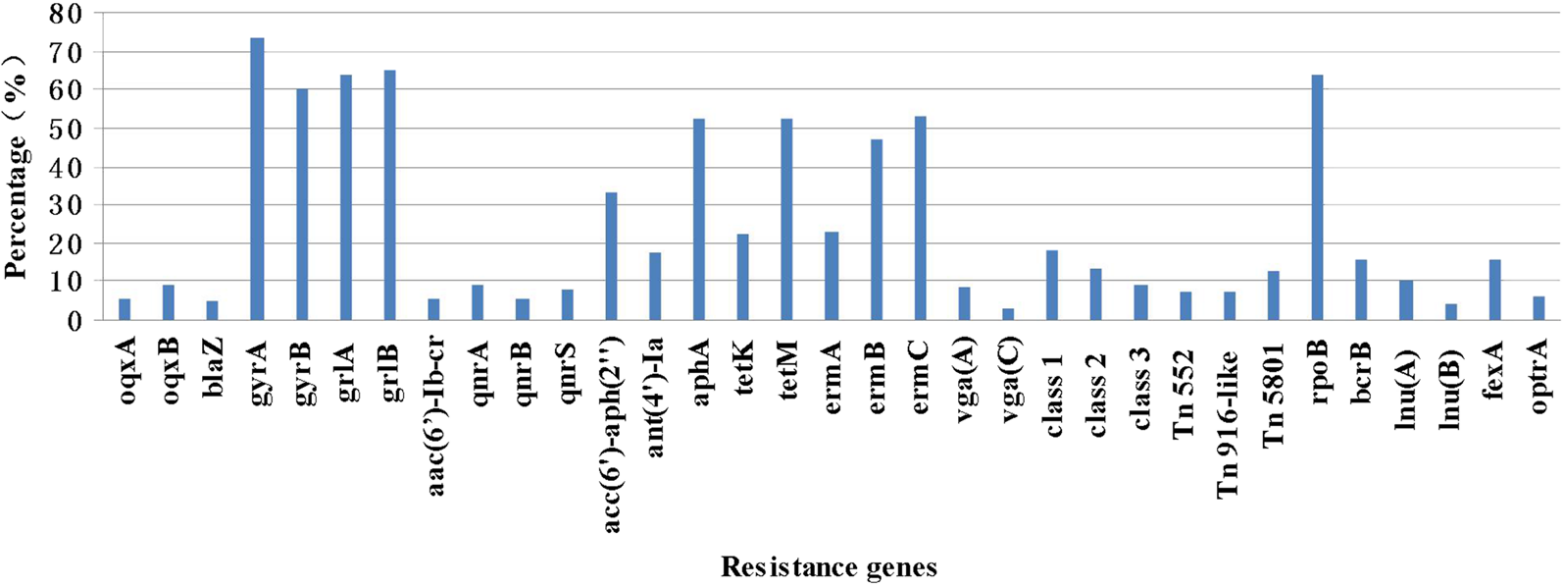
Percentage of positive resistance genes in *Staphylococcus aureus* isolates. The graph’s horizontal axis shows the each of resistance genes, and the vertical axis shows the percentages

The distribution of resistance genes between the different *S. aureus* sources is shown in Additional file 1: Table S1 and Fig. 1. A total of 143 *S. aureus* isolates were tested for 46 resistance genes. Of these genes, 34 (73.91%, 34/46) were detected, and 12 (26.09%, 12/46), undetected. Overall, tetracycline resistance was reported in 143 *S. aureus* isolates, probably owing to the presence of the membrane-associated efflux gene *tetK* (32, 22.38%) or the ribosome-binding site gene *tetM* (75, 52.45%). Aminoglycosides resistance genes *acc(6′)*-*aph(2″)*, *ant(4′)*-*Ia*, and *aphA* were detected in 46 (32.87%), 25 (17.48%), and 75 (52.45%) isolates, respectively. Macrolides resistance genes *ermA*, *ermB*, and *ermC* were detected in 33 (23.08%), 67 (46.85%), and 76 (53.15%) isolates, respectively, while lincosamides resistance genes *lnu(A)* and *lnu(B)* were detected in 15 (10.49%), and 6 (4.20%) isolates, respectively, *lnu(C)* and *lnu(D)*, were undetected. Streptogramins, lincosamides, and pleuromutilin resistance genes *vga(A)* was detected in 12 (8.39%) isolates, while only four isolates carried *vga(C)*. Olaquindox resistance was related to the associated efflux genes *oqxA* (8, 5.59%) and *oqxB* (13, 9.09%). The integrase genes of class I, II, and III were detected in 26 (18.18%), 19 (13.29%), and 13 (9.09%) isolates, respectively. Resistance genes for fluoroquinolones and mutations (*aac*(*6′*)-*Ib*-*cr*, *qepA*, *qnrA*, *qnrB*, *qnrC*, *qnrD*, *qnrS*, *gyrA*, *gyrB*, *grlA and grlB*) are summarized in Additional file 1: Table S1.

Of all 143 *S. aureus* isolates, 7 (4.90%) isolates carried β-lactamase gene *blaZ*, 91 (63.64%) isolates were positive for the rifampicin resistance gene *rpoB*, and 22 (15.38%) carried the chloramphenicol and florfenicol resistance gene *fexA*. In addition, nine (6.29%) isolates had the oxazolidinone resistance gene *optrA*, while the bacitracin resistance gene *bcrB* was detected in 22 (15.38%) isolates. However, the vancomycin resistance gene vanA, fosfomycin resistance gene *fosB*, chloramphenicol and florfenicol resistance gene *cfr*, and bacitracin resistance genes (*bcrA*, *bcrD*, and *bcrR*) were absent in all isolate.

### Prevalence of virulence genes

**Fig. 2:**
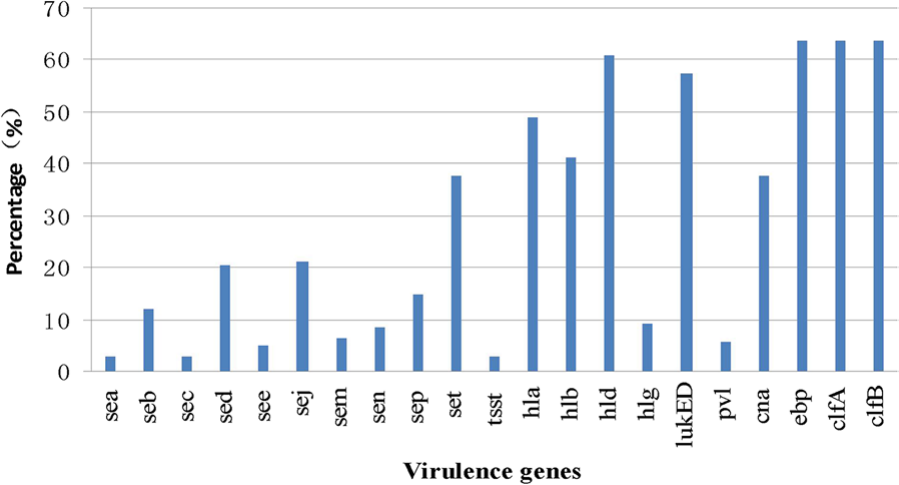
Percentage of positive virulence genes in *Staphylococcus aureus* isolates. The graph’s horizontal axis shows the each of virulence genes, and the vertical axis shows the percentages

The distribution of virulence genes between the different *S. aureus* sources is shown in Additional file 1: Table S1 and Fig. 2. A total of 143 *S. aureus* isolates were tested for 24 virulence genes. Among these genes, 21 (87.50%, 21/24) were detected, and 3 (12.50%, 3/24), undetected. A more diverse range of enterotoxin genes were detected among these isolates, including *sea* (4, 2.80%), *seb* (17, 11.89%), *sec* (4, 2.80%), *sed* (29, 20.28%), *see* (7, 4.90%), *sej* (30, 20.98%), *sem* (9, 6.29%), *sen* (12, 8.39%), *sep* (21, 14.69%) and *set* (54, 37.76%).

As shown in Additional file 1: Table S1, the *TSST*-*1* virulence gene was detected in only four (2.80%) isolates. The β-hemolysin genes *hla*, *hlb*, *hld*, and *hlg* were identified in 70 (48.95%), 59 (41.26%), 87 (60.84%), and 13 (9.09%) isolates, respectively. The Panton-Valentine leukocidin (*PVL*) virulence genes were tested among these isolates, and the majority of the strains carried the *lukED* gene (82, 57.34%), while a small number of isolates carried *pvl* gene (8, 5.59%). However, the adherence factor gene *cna* was detected in 54 (37.76%) isolates and all of genes *ebp*, *clfA*, and *clfB* were detected in 91 (63.64%) isolates. All the isolates were negative for the exfoliative toxin genes *eta*, *etb*, and *etd*. A high prevalence of virulence genes *ebp*, *clfA*, *clfB*, *hla*, *hld*, and *lukED* was observed in 143 *S. aureus* isolates.

### Genomic macrorestriction and PFGE typing

**Fig. 3:**
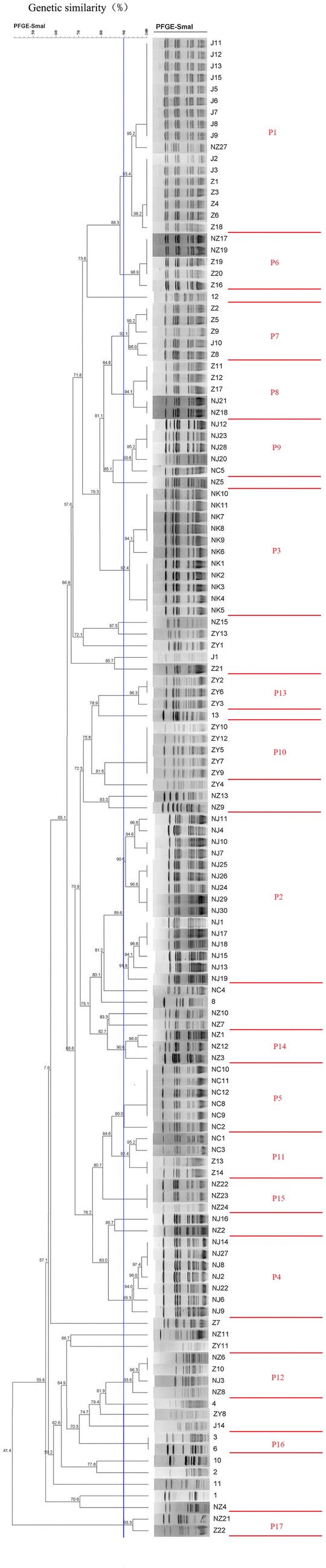
Dendrogram of PFGE profiles of *Sma* I-digested Genomic DNA of genetically unrelated *S. aureus* isolates. Similarities percentage is identified on a dendrogram derived from the unweighted pair group method using arithmetic averages and based on Dice coefficients. The vertical blue line shows the 90% similarity cut-off, whilst the pulsotypes are delineated by red lines

We performed PFGE-typing for all 143 *S. aureus* isolates, and only 130 (82.52%) isolates could be typed using DNA macrorestriction followed by PFGE with *Sma I* digestion. These 130 *S. aureus* isolates revealed 45 different electrophoretic patterns (P) with homologies between 41.4% and 100%. These were grouped into 17 pulsotypes (P1–P17) and 28 separate pulsotypes using a 90% cut-off value (Fig. 3). The analysis of 130 isolates that were grouped into 10 major pulsotypes designated as P1 (17/130), P2 (15/130), P3 (11/130), P4 (7/130), P5 (6/130), P6 (5/130), P7 (5/130), P8 (5/130), P9 (5/130), and P10 (5/130) accounted for 62.3% of these isolates. The two main clusters P1 and P2 contained 17 and 15 isolates, respectively.

Two PFGE pulsotypes, P7 and P12, were detected in all three geographic regions, while PFGE pulsotypes P18, P16, and P17, each with only one or two isolates, were more unevenly distributed (Fig. 3). Strains isolated from swine, as well as from the chickens or raw milks were present in these difference clusters, suggestive of the possible transmission through the swine farm or slaughtering procedure (Fig. 3). Identical the same PFGE patterns were observed for strains from swine, chicken, and raw milk, indicating that the infection was probably acquired through the spread of *S. aureus*.

### *Spa* typing

*Spa* typing was performed for all 143 *S. aureus* isolates. Only 118 (82.52%) isolates were successfully confirmed and these were assigned to 26 *spa* types, which varied in length between 1 and 10 repeat units (Table 4).

**Table 4:** Prevalence of the *spa* type among the *S. aureus* isolates

Spa types	Spa repeats	Repeat units	Strain number	Total (%)
t030	r15-r12-r16-r02-r24-r24	6	ZY2, ZY3, ZY4	3 (2.10)
t034	r08-r16-r02-r25-r02-r25-r34-r24-r25	9	Z9, Z11, Z14, Z15, J1, J4, J10, NC6, NC8, NC10, NC11, NC12	12 (8.39)
t091	r07-r23-r21-r17-r34-r12-r23-r02-r12-r23	10	Z2, Z8, Z10, Z13, Z17, Z21, NC1, NC2, NC3, NC4	10 (7.00)
t127	r07-r23-r21-r16-r34-r33-r13	7	NJ1, NJ7, NJ10, NJ11, NJ13, NJ18, NJ19, NJ24, NJ29	9 (6.29)
t164	r07-r06-r17-r21-r34-r34-r22-r34	8	NZ3, NZ9, NZ11, NZ15, NZ20, NZ24, NZ25, NZ26	8 (5.60)
t189	r07-r23-r12-r21-r17-r34	6	NK1, NK2, NK4, NK5, NK7, NK9, NK10, NK11, NJ17, NZ1, NZ5, NZ12, NZ27, 1, 3, 4, 5, 10	18 (12.59)
t267	r07-r23-r12-r21-r17-r34-r34-r34-r33-r34	10	NJ9, NJ16, 13	3 (2.10)
t437	r04-r20-r17-r20-r17-r25-r34	7	ZY1	1 (0.70)
t458	r26	1	NJ2, NJ8, NJ14, NJ22, NJ23, NJ28	6 (4.20)
t605	r07-23	2	NJ6	1 (0.70)
t693	r07	1	NJ15	1 (0.70)
t730	r07-r34-r34-r34-r33-r34	6	NZ2	1 (0.70)
t865	r07-r23-r12-r21-r17-r34-r34-r34	8	NJ25	1 (0.70)
t899	r07-r16-r23-r02-r34	5	Z7, NC5	2 (1.40)
t2193	r07-r12-r21-r17-r13-r34-r34	7	ZY11	1 (0.70)
t2224	r07-r23-r12-r21-r17-r34-r34-r34-r33-r13	10	12	1 (0.70)
t2646	r26-r17-r34-r17-r17-r16	6	J2	1 (0.70)
t3155	r07-r12-r21-r17-r13-r34-r34-r33-r13	9	ZY6, ZY7, ZY9, ZY10, ZY12	5 (3.50)
t3380	r07-r23-r12-r21-r17-r34-r34	7	NJ26, NZ10	2 (1.40)
t3512	r07-r16-r23-r02	4	Z12, NC7, 8, 9, 11	5 (3.50)
t3527	r04-r20-r17-r02-r17-r25-r34	7	ZY13	1 (0.70)
t3626	r07-r23-r12-r21-r17-r34-r34-r34-r33	9	NJ4	1 (0.70)
t6367	r07-r23-r12-r21-r17	5	NK8, NZ7	2 (1.40)
t6811	r07-r23-r21-r16-r34-r33	6	NJ30	1 (0.70)
t8139	r07-r23-r12-r21-r17-r17	6	NK3, NZ22	2 (1.40)
t15075	r26-r17-r34-r17-r17-r17-r16	7	Z1, Z3, Z4, Z5, Z6, Z16, Z18, Z19, Z20, Z22, J3, J5, J6, J7, J8, J9, J11, J12, J13, J15	20 (14.00)
no *spa*^a^	–	–	J14, NK6, NC9, ZY5, ZY8, NJ3, NJ5, NJ12, NJ20, NJ21, NJ27, NZ4, NZ6, NZ8, NZ13, NZ14, NZ16, NZ17, NZ18, NZ19, NZ21, NZ23, 2, 6, 7	25 (17.48)

^a^Not available

Among the 118 *S. aureus* isolates, a diverse range of *spa* types were detected; t15075 (20/118, 14.00%) was the most common type, followed by t189 (18/118, 12.59%), t034 (12/118, 8.39%), t091 (10/118, 7.00%), t127 (9/118, 6.29%), and t164 (8/118, 5.60%). However, only t034 (12/118, 8.39%) was present in isolates from the three animals sources. The distribution of the 118 *S. aureus spa* types are correlated with the repeat units (Table 4).

### SCC*mec* typing

Of the eight MRSA isolates, SCC*mec* was detected in seven isolates. The most prevalent type was SCC*mec* IVa (n = 3), followed by SCC*mec* III (n = 2), SCC*mec* II (n = 1), and SCC*mec* I (n = 1). One MRSA isolates could not be SCC*mec* typed.

## Discussion

In this study, we investigated the prevalence and performed molecular characterization of *S. aureus* isolated from Henan province, China, to facilitate better understanding of the epidemiology of *S. aureus*. We found that the prevalence of *S. aureus* strains was 22.3%, consistent with the previous reports [14], but was significantly higher than that reported in other countries [15, 16]. Unlike a previous study [1] where in all *S. aureus* samples were collected from animals and humans, more than 90% of the 143 *S. aureus* isolates in the present study were recovered from healthy animals. The percentage of animal samples positive for *S. aureus* (23.7%) in our study was similar to that previously reported [17]. However, some previous studies have detected higher percentages [18], while others have reported lower percentages [11, 12]. The percentage of human samples detected positive for *S. aureus* (n = 92, 13 isolates, 14.1%) in our study was similar to that reports in a previous study [19], but other studies have detected higher percentages [20] and some studies have reported lower percentages [21]. In addition, none of the 44 samples from the Henan Province People’s Hospital was found positive, probably owing to the difference in the nature or source of samples. Hence, further studies are warranted.

In the present study, the prevalence of MRSA was only 5.59%. MRSA is known to cause a wide variety of infections in humans and animals. Very little is known about the frequency of MRSA transmission between animals and humans,but MRSA transmission from healthy animals poses a great threat to medical science and veterinarian clinic. As observed with *S. aureus*, MRSA may cause some infections in humans and animals. The percentages of MRSA detected in other studies have been variable [20–22]. Studies on the prevalence of MRSA in China have detected MRSA isolates in animal and human samples [1, 23]. MRSA prevalence in China was 27.5% in 1999 and rapidly reached 60.7% in 2009 [24]. Other studies have reported 69.5% and 78.5% MRSA prevalence in Shanghai and Guangzhou, respectively. Inland cities such as Chongqing have been reported to exhibit a prevalence of 45.0% [25]. In the present study, *Cap*5 (56.64%) was detected as the dominant serotype. However, *Cap*8 has been reported as the dominant serotype in other studies [26].

In this study, we observed high resistance rates. The resistance rates to penicillin (96.50%) and tetracycline (68.53%) detected in this study were similar to those observed in previous studies [15, 27]. The high resistance rates may be related to the use of antimicrobials treating mastitis in cattle farm and growth promotion or prophylaxis in swine and chicken. The prevalence of resistance to multiple antibiotics detected in our study was similar to that reported in previous studies [28]. In addition, the antibiotic resistance patterns of *S. aureus* strains recovered from raw milk and dairy products were reported [3]. In our study, oxacillin resistance was detected in strains isolated from raw milk, chicken, and human isolates, in contradiction with the previous reports [1, 29]. These studies reported that MRSA are only found in pig farms but not in raw milk, chicken, and human isolates. Furthermore, 8 *mecA*-positive isolates were resistant to oxacillin. Previously reported a significant correlation between oxacillin resistance and resistance to ciprofloxacin, clindamycin, gentamycin, and erythromycin [30], consistent with the results of our study. In addition, some *S. aureus* isolates were resistant to methicillin, but lack of *mecA* gene, as reported in another study [31]. Therefore, phenotypically resistant MRSA could be misdiagnosed using molecular methods alone, suggestive of the multiple mechanisms in MRSA. In addition, tigecycline, showed good activity against *S. aureus*, thereby ensuring effective control efforts in humans. Tigecycline resistance was recently identified for different pathogens, especially in MDR strains [32]. Hence, further studies are needed to evaluate the transmission between animals and humans.

Identification of the *mecA* gene in *S. aureus* is a gold standard for the detection of MRSA, which exhibits low affinity for β-lactam antimicrobials [18]. The *blaZ* gene confers penicillin resistance. We found that 96.50% of strains were resistant to penicillin, but only eight (4.90%) strains harbored *blaZ*. This observation is in line with the previously reported results [33]. In our study, both *tetM* and *tetK* were detected in tetracycline-resistant strains, whereas approximately 80% of tetracycline-resistant strains carried *tetM*, as previously observed [33]. In addition, the majority of the strains carried *tetM* as well as *blaZ*, consistent with the results of a previous study [34]. Macrolide resistance genes *ermA*, *ermB*, and *ermC* were present either alone or in combination with *erm*(*A*)+ *erm*(*B*), *erm(B) *+ *erm(C)*, or *erm(A) *+ *erm(B) *+ *erm(C)*, which was in accordance with a previous study [35]. In this study, 22 (15.38%) isolates carried florfenicol resistance gene *fexA*, and the number was significantly higher than that reported in a previous study [36]. Gentamicin resistance was associated with the aminoglycoside resistance genes *acc(6′)*-*aph(2″)*, *ant(4′)*-*Ia*, or *aphA*, and these three genes co-existed in the majority of isolates, as observed in a previous study [35]. The location of *vga(A)* or *vga(C)* on a plasmid may play an important role in its persistence and dissemination [22, 37]. Bacitracin, a polypeptide antibiotic, is used as an animal growth promoter for prophylaxis and therapy of consumable animals in China. In the present study, the prevalence of the *bcrB* gene in swine strains (59.1%) was higher than that in chicken strains (46.7%); this observation may be largely related to the wider use of bacitracin as a feed additive in swine than in chicken. To the best of our knowledge, this is the first report to demonstrate *bcrB* gene in *S. aureus* isolates from animals in Henan province, China. Some recent reports have shown the widespread of the high-level bacitracin resistance (MIC ≥ 256 μg/mL) in enterococci [38, 39]. In the present study, the prevalence of linezolid resistance gene *optrA* in chicken strains (26.7%) was higher than that in swine strains (13.6%). This is the first report to detect *optrA* gene in *S. aureus* isolates from animals in Henan province, China. The gene *optrA* was recently detected in *S. sciuri* from swine [40]. Although linezolid is not approved for use in animals, selective pressure from other antibiotics such as florfenicol, tiamulin, and lincomycin that are widely used in animals may promote the spread of *optrA*. Thus, more attention needs to be paid to the possibility that *optrA* may find its way through the food chain or pathogenic bacteria of humans. In addition, the emergence and dissemination of these MDR isolates in animals pose a threat to public health, given that *optrA*-mediated linezolid resistance may rapidly spread among different bacterial species. Therefore, the surveillance of *optrA* gene in China is very important to limit its dissemination to prevent the potential threat to animal and human health.

The existence of SEs in *S. aureus* isolated from animals and humans vary from our reported. The results of the present study on the existence of *sea*, *seb*, and *sec* in all *S. aureus* isolates are contradictory to those previously reported, wherein *sea* was observed in 45.2% of isolates and *seb* was detected in 18.5% of isolates [41]. In another study, *sea* was reported in 26.2% of isolates and *seb* in 39.3% of isolates [23]. However, the detection rate for *sec* gene was low in 6% of the isolated *S. aureus* strains [42]. The high occurrence of β-hemolysin genes *hla* (48.95%) and *hlb* (41.26%) among *S. aureus* isolates is in line with the results of other reports [43]. In our study, the *TSST* virulence gene associated with *TSST*-*1* was detected in only 4 (2.80%) isolates, consistent with the results of another study [41]. Moreover, more than half of the strains carried *lukED* gene (57.34%), as previously reported [19]. In addition, a small number of isolates carried *pvl* gene (8, 5.59%), consistent with a previous report [23], and this number was lower than the occurrences of *pvl*-positive *S. aureus* in a previous study [44]. In the present study, four of *pvl*-positive isolates were MRSA, while the remaining four isolates showed different molecular types.

The results of PFGE analysis showed that a part of the isolates were identical and showed the same PFGE patterns (P1). These were derived from swine, chicken, and raw milks of different animals. This observation is in line with that reported in a previous study, wherein same PFGE patterns were observed for strains from goat milk powders at different processing stages [3]. Thus, cross contamination of *S. aureus* may occur in different animals. A previous study showed that each region had its own predominant PFGE pattern [22]. Identical the same PFGE patterns were observed for strains from swine, chicken, and raw milks of different animals, supporting our hypothesis that the infection may be probably acquired through the spread of *S. aureus*. Strains isolated from swine and chicken or raw milks were present in these clusters, suggestive of the possible transmission during the slaughtering procedure as previously reported [15]. As observed in the *spa* typing results, t15075 and t189 were the most common *spa* types. However, *t899* was the most prevalent *spa* types in *S. aureus* [22, 45]. Although PFGE patterns of the isolates showed more variations than those observed by *spa* typing, new technologies such as next generation sequencing may provide better understanding of the origin, transmission, and evolution of MRSA. These advanced technologies would be included in our further studies on the origin and spread of MRSA in China [22]. In this study, PFGE and *spa* typing were used in combination with *SCCmec* typing. The majority of the strains were assigned to ten major PFGE types, and P1 and P2 were the two most common clusters containing 17 and 15 isolates, respectively. Moreover, PFGE type P1 among three *spa* types (t15075, t189, and t2646), *spa* types t189 among main three clusters (P1, P2, and P3). In this study, MRSA-SCC*mec* Iva-t437 was observed in human isolates, as previously reported [23, 46].

## Conclusion

In conclusion, this study presents the first insight into the prevalence, antimicrobial resistance, virulence factors, and molecular characterization of *S. aureus* isolates from healthy animals and patients in Henan Province, China. The high prevalence of *S. aureus* highlights the importance of effective animal hygiene measures to prevent the further spread of *S. aureus*. It is important to consider the prevalence of *S. aureus* in raw milk and the risk of its transmission through the food chain. Moreover, high resistance rates were observed, necessitating strict supervision. Future epidemiological investigations should be conducted with larger number of strains and samples.

## Methods

### Collection of samples

From September 2013 to June 2014, a total of 640 samples, including 548 animal samples, and 92 patient samples, were collected in Henan province, China. We collected 548 samples (cows, n = 350; swine, n = 86; chickens, n = 70; ducks, n = 42) from healthy animals in large-scale farms. In addition, 92 patient samples from various clinical specimens were obtained from the First Affiliated Hospital of Zhengzhou University (n = 48) and Henan Province People’s Hospital (n = 44) in Zhengzhou city. These specimens were recovered from adult humans with symptoms of pneumonia, diarrhea and pyogenic infection. The samples were transported to the laboratory under required preservation conditions (in a cooler with ice) within 6 h of collection and processed within 2 h.

### Isolation and identification of *S. aureus*

Isolation and identification of *S. aureus* were performed by enrichment and sequential plating onto selective plates, as previously described [47]. The samples were incubated in brain–heart infusion (BHI) (Beijing Land Bridge Technology Co., Ltd, China) broth containing 7.5% sodium chloride (NaCl) at 37 °C overnight. Colonies were purified on tryptone soy agar (TSA) plates (Beijing Land Bridge Technology Co., Ltd, China). The broth was streaked onto CHROMagar *S. aureus* (CHROMagar, France) plates and incubated at 37 °C for 24 h to obtain presumptive isolates of *S. aureus*. These presumptive isolates were identified as *S. aureus* following by Gram staining, and catalytic reactions using VITEK-2 compact automated identification system (BioMérieux, Marcy-I’Etoile, France). *S. aureus* ATCC 29213 was used as a positive reference strain.

### Molecular identification of thermonuclease (*nuc*) and *mecA* genes

Bacterial genomic DNA was obtained from 2 mL of the bacterial cell suspension incubated overnight in BHI. Single colonies were grown on BHI agar and transferred to 2 mL of BHI broth. Then cultures were centrifuged for 8 min at 12,000×*g*, and the DNA pellets were resuspended in 50 μl of lysostaphin solution (30 mg/mL; Shanghai Yuanye Bio-Technology Co., Ltd, China). After 10 min of incubation at 37 °C, 50 μl of Proteinase K (100 mg/L) and 100 μl of TE buffer (10 mM Tris–HCl buffer [pH 7.5] and 1 mM ethylenediaminetetraacetic acid [EDTA]) were added; the sample was mixed by vortexing and incubated for 10 min at 37 °C. The sample were heated in a thermocycler at 99.9 °C for 10 min and immediately incubated at − 20 °C for 15 min, followed by centrifugation at 12,000×*g* for 15 min. A total of 100 μl of supernatant was removed without disturbing the pellet and dissolved in water, and the extracted genomic DNA was stored at − 20 °C as a PCR template.

The identity of *S. aureus* isolates were further confirmed by PCR using a species-specific primer (F: 5′-GCGATTGATGGTGATACGGTT-3′, R: 5′-AGCCAAGCCTTGACGAACTAAAGC-3′) that codes for *nuc*, as previously described [48]. *S. aureus* (ATCC 29213) was used as a positive control. The identified *S. aureus* isolates were confirmed as MRSA by amplifying the methicillin resistance-encoding *mecA* gene with *mecA* primers (F: 5′-TCCAGATTACAACTTCACCAGG-3′, R: 5′-CCACTTCATATCTTGTAACG-3′) [49].

### Identification of *S. aureus Cap*5 and *Cap*8

The serovars of *S. aureus* isolates were determined by *Cap* tests, *Cap*5 and *Cap*8 were detected according to the previously described method [26].

### Antimicrobial susceptibility testing

The susceptibility of 143 *S. aureus* to 33 antibiotics in this study was tested (Additional file 2: Table S2) by the broth microdilution method according to the Clinical and Laboratory Standards Institute (CLSI) guidelines [50]. MICs for penicillin, methicillin, oxacillin, ceftriaxone, ceftiofur, cefquinome, cefepime, imipenem, cefoperazone and sulbactam sodium (2:1), piperacillin and tazobactam sodium (4:1), ciprofloxacin, enrofloxacin, levofloxacin, gentamicin, amikacin, neomycin, chloramphenicol, florfenicol, tylosin, erythromycin, Lincomycin, tetracycline, terramycine, doxycyclin, tigecycline, olaquindox, mequindox, fosfomycin, rifampicin, vancomycin, bacitracin, antimicrobial peptide, and linezolid were determined.

We used *S. aureus* ATCC 29213 as a reference strain for quality control in MIC determinations. Results were interpreted in accordance with the CLSI guidelines [50]. If CLSI criteria were unavailable for some antibiotics, results were interpreted according to criteria of the European Committee on Antimicrobial Susceptibility Testing (EUCAST) [51].

### Determination of antimicrobial resistance genes and integrons

Resistance genes for olaquindox (*oqxA* and *oqxB*), β-lactams (*blaZ*), vancomycin (*vanA*), fosfomycin (*fosB*), rifampicin (*rpoB*), oxazolidinone (*optrA*), chloramphenicol and florfenicol (*cfr* and *fexA*, respectively), fluoroquinolones and mutations (*aac(6′)*-*Ib*-*cr*, *qepA*, *qnrA*, *qnrB*, *qnrC*, *qnrD*, *qnrS*, *gyrA*, *gyrB*, *grlA*, and *grlB*), aminoglycosides (*acc(6′)*-*aph(2″)*, *ant(4′)*-*Ia*, and *aphA*), tetracycline (*tetk* and *tetM*), macrolides (*ermA*, *ermB*, and *ermC*), streptogramins, lincosamides and pleuromutilin (*vga(A)*, *vga(B)*, and *vga*(*C*)), lincosamides (*lnuA*, *lnuB*, *lnuC*, and *lnuD*), bacitracin (*bcrA*, *bcrB*, *bcrD*, and *bcrR*), integrase genes of *class I*, *II*, and *III* were determined by PCR. The primers used and amplicon band sizes are shown in Additional file 3: Table S3. All PCR products were sequenced and compared with sequences in National Center for Biotechnology Information (NCBI; http://www.ncbi.nlm.nih.gov).

### Prevalence of virulence genes

A total of 143 *S. aureus* isolates were screened for 24 different virulence genes from seven different toxin gene groups, including enterotoxins (*sea*, *seb*, *sec*, *sed*, *see*, *sej*, *sem*, *sen*, *sep*, and *set*), hemolysins (*hla*, *hlb*, *hld*, and *hlg*), leucocidin (*lukED*), Panton-Valentine leucocidin (*pvl*), adherence factors (*cna*, *ebp*, *clfA*, and *clfB*), toxic shock syndrome toxin (*TSST*), and exfoliative toxins (*eta*, *etb*, and *etd*). The primers and amplicon band sizes are listed in Additional file 4: Table S4. At least one representative PCR amplicon of each positive virulence gene was sequenced using the same primers to confirm the accuracy of the PCR amplification.

### Pulsed-field gel electrophoresis typing

We performed PFGE typing according to the CDC standard protocol developed by PulseNet for *S. aureus* [52]. Genomic DNA of all 143 *S. aureus* isolates were prepared from 5 mL *S. aureus* culture grown overnight in Luria–Bertani (LB) broth (Beijing Land Bridge Technology Co., Ltd, China). Briefly, agarose-embedded DNA was digested using the restriction enzyme *Sma I* (TaKaRa, Dalian) for 6 h in a water bath at 30 °C. Restriction fragments of DNA were separated by PFGE using 1.0% Seakem Gold Agarose gels (Lonza, USA) with the PFGE apparatus CHEF Mapper electrophoresis system (Bio-Rad) at 6 V/cm and 14 °C in 0.5× TBE buffer for 19 h with the pulse time ranging from 5.2 to 41 s at an angle of 120°. After electrophoresis, gels were stained with ethidium bromide and photographed with Gel Doc XR digital imaging system (Bio-Rad) under UV illumination and stored as TIFF files. Gel images were analyzed using BioNumerics Software (version 4.0, Applied Maths, Kortrijk, Belgium). The banding patterns in different gels were clustered using Dice coefficients with 1.4% band tolerance and 1% optimization settings, and unweighted pair group method with arithmetic mean (UPGMA) was used to calculate Dice coefficients of correlation. This result corresponded to a similarity coefficient of 90% was used to define a PFGE cluster (pulsotype, P). *Salmonella* Branderup strain H9812 was digested with *Xba I* (TaKaRa, Dalian) and used as a molecular size marker.

### *Spa* typing

*Spa* typing was based on variations of the repeat units; the *spa* gene from the X region was amplified by PCR [53]. Primers *spa*-1113f (5′-TAAAGACGATCCTTCGGTGAGC-3′) and *spa*-1514r (5′-CAGCAGTAGTGCCGTTTGCTT-3′) were used for amplification (http://www.ridom.de/doc/Ridom_spa_sequencing.pdf). We assigned *spa* typing by submitting the data to the SpaServer Database (http://spaserver.ridom.de). The software Ridom StaphType (Ridom GmbH, Würzburg, Germany) was used for *spa* sequence analysis.

### *Staphylococcus* cassette chromosome *mec* typing

*Staphylococcus* cassette chromosome *mec* typing was performed using multiplex PCR schemes, as per a previously reported method [54]. MRSA isolates that showed unrelated patterns or could not be assigned to any expected type were defined as non-typable [55].

### Data analysis and statistical methods

All 143 isolated strains were categorized as sensitive (S) or resistant (R) based on the MIC values. The MIC range, MIC_50_, and MIC_90_ were analyzed. For statistical analysis, Chi square test was performed, and *p* values of ≤ 0.05 were considered statistically significant, and 95% confidence intervals were calculated using SPSS 20.0 software (IBM, USA).

## Additional files

**Additional file 1: Table S1.** Characteristics of the 143 *S. aureus* isolates.

**Additional file 2: Table S2.** A list of the 33 tested antimicrobials, their classes, and the concentrations used for susceptibility testing of *S. aureus.*

**Additional file 3: Table S3.** Oligonucleotide sequences, primers, and targets for polymerase chain reaction amplification of antibiotic resistance genes and integrons in *S. aureus* isolates.

**Additional file 4: Table S4.** Oligonucleotide sequences, primers, and targets for polymerase chain reaction amplification of virulence genes in *S. aureus* isolates.

## Abbreviations

*S. aureus*: *Staphylococcus aureus*
MRSA: methicillin-resistant *S. aureus*
FDA: food and drug administration
MDR: multidrug resistant
CLSI: Clinical and Laboratory Standards Institute
MICs: minimum inhibitory concentrations
EUCAST: European Committee on Antimicrobial Susceptibility Testing
SCC*mec*: *Staphylococcus* cassette chromosome *mec*
