# Supplementary material for: Prevalence, resistance pattern, and molecular characterization of Staphylococcus aureus isolates from healthy animals and sick populations in Henan Province, China
Source: Gut Pathog. 2018 Jul 17;10:31. doi: 10.1186/s13099-018-0254-9 (PMC6048774; doi:10.1186/s13099-018-0254-9)
Supplement: Supplementary file 1 — Additional file 1: Table S1. Characteristics of the 143 S. aureus isolates. [file 13099_2018_254_MOESM1_ESM.pdf]

Table S1. Characteristics of the 143 *S. aureus* isolates

Strain	Source	Serotype	Resistance pattern	Resistance gene and integron	Virulence gene	PFGE	spa	SCCmec
NJ1	raw milk	cap8	八	gyrA-gyrB-grlA-grlB-tetM-ermC-rpoB	set-hla-hlb-hld-lukED-can-ebp-clfA-clfB	P20	t127	
NJ2	raw milk	cap5	八	gyrA-gyrB-grlA-grlB-tetM-ermC-rpoB	hla-hlb-hld-lukED-ebp-clfA-clfB	P31	t458	
NJ3	raw milk	*	九	-	-	P35	-	
NJ4	raw milk	cap8	十	gyrA-gyrB-grlA-grlB-tetM-ermC-rpoB-class I	hla-hlb-hld-lukED-can-ebp-clfA-clfB	P20	t3626	
NJ5	raw milk	*	十一	oqxA-oqxB-gyrA-qnrS	set	-	-	
NJ6	raw milk	cap5	九	gyrA-gyrB-grlA-grlB-qnrB-tetM-ermC-rpoB	set-hla-hlb-hld-lukED-ebp-clfA-clfB	P31	t605	
NJ7	raw milk	cap8	十	gyrA-gyrB-grlB	set-hla-hlb-hld-lukED-can-ebp-clfA-clfB	P20	t127	
NJ8	raw milk	cap5	八	gyrA-gyrB-grlA-grlB-aphA-tetM-ermC-rpoB	set-hla-hlb-hld-lukED-ebp-clfA-clfB	P31	t458	
NJ9	raw milk	cap5	十	gyrA-gyrB-grlA-grlB-aphA-tetK-tetM-ermC-rpoB	hla-hlb-hld-lukED-ebp-clfA-clfB	P31	t267	
NJ10	raw milk	cap8	八	gyrA-gyrB-grlA-grlB-aphA-tetM-ermC-rpoB	set-hla-hlb-hld-lukED-can-ebp-clfA-clfB	P20	t127	
NJ11	raw milk	cap8	九	gyrA-gyrB-grlA-grlB-aphA-tetM-ermC-rpoB-Tn 552	Set-hla-hlb-hld-lukED-can-ebp-clfA-clfB	P20	t127	
NJ12	raw milk	*	六	acc(6')-aph(2″)-aphA-tetM-ermB-lnu(B)	-	P6	-	
NJ13	raw milk	cap8	八	gyrA-gyrB-grlA-grlB-ermC-rpoB	set-hla-hlb-hld-lukED-can-ebp-clfA-clfB	P20	t127	
NJ14	raw milk	cap5	九	gyrA-gyrB-grlA-grlB-tetM-ermC-rpoB	hla-hlb-hld-lukED-ebp-clfA-clfB	P31	t458	
NJ15	raw milk	cap5	五	-	-	P20	t693	
NJ16	raw milk	cap5	十	gyrA-gyrB-grlA-grlB-aphA-tetK-tetM-ermB-ermC-rpoB	set-hla-hlb-hld-lukED-ebp-clfA-clfB	P29	t267	
NJ17	raw milk	cap8	六	gyrA-gyrB-grlA-grlB-acc(6')-aph(2″)-ermC	set-hla-hlb-hld-lukED-can-ebp-clfB	P20	t189	
NJ18	raw milk	cap8	七	gyrA-gyrB-grlA-grlB-ermC-rpoB	set-hla-hlb-hld-lukED-can-ebp-clfA-clfB	P20	t127	
NJ19	raw milk	cap8	七	ermC	hla-hlb-hld-lukED	P20	t127	
NJ20	raw milk	*	四	qnrB-acc(6')-aph(2″)-aphA-tetM-ermB-lnu(B)	lukED	P6	-	
NJ21	Raw milk	*	八	oqxA-oqxB-acc(6')-aph(2″)-aphA-tetM-ermB-ermC-class I	-	P5	-	
NJ22	raw milk	cap5	八	gyrA-gyrB-grlA-grlB-ermC-rpoB	set-hla-hlb-hld-ebp-clfA-clfB	P31	t458	
NJ23	raw milk	cap5	七	gyrA-grlB-ermC	seb-set-hla-hlb-hld-lukED-pvl-can-ebp-clfA-clfB	P6	t458	
NJ24	raw milk	cap8	八	gyrA-gyrB-grlA-grlB-ermC-rpoB	set-hla-hlb-hld-lukED-can-ebp-clfA-clfB	P20	t127	
NJ25	raw milk	cap8	六	gyrA-gyrB-grlA-grlB-ermC-rpoB	set-hla-hlb-hld-lukED-can-ebp-clfA-clfB	P20	t865	
NJ26	raw milk	cap8	八	gyrA-gyrB-grlA-grlB-ermC-rpoB-Tn 552	sea-set-hla-hlb-hld-hlg-lukED-pvl-can-ebp-clfA-clfB	P20	t3380	
NJ27	raw milk	*	八	acc(6')-aph(2″)	-	P31	-	
NJ28	raw milk	cap5	七	gyrA-gyrB-grlA-grlB-aphA-tetM-ermB-ermC-rpoB	set-hla-hlb-hld-lukED-ebp-clfA-clfB	P6	t458	
NJ29	raw milk	cap8	七	gyrA-gyrB-grlA-grlB-ermC-rpoB	set-hla-hlb-hld-lukED-can-ebp-clfA-clfB	P20	t127	
NJ30	raw milk	cap8	六	gyrA-gyrB-grlA-grlB-aphA-tetM-ermB-ermC-rpoB	set-hla-hlb-hld-lukED-can-ebp-clfA-clfB	P20	t6811	
NZ1	raw milk	cap8	三	gyrA-gyrB-grlA-grlB-ermC-rpoB-lnu(A)	hlb-lukED-can-ebp-clfA-clfB	P25	t189	
NZ2	raw milk	cap5	八	oqxA-gyrA-gyrB-grlA-grlB-rpoB-class II	sec-sed-sej-tsst-hla-hlb-hld-lukED-ebp-clfB	P30	t730	
NZ3	raw milk	cap8	六	tetM	hlb-hld-lukED-can-ebp-clfA-clfB	P25	t164	
NZ4	raw milk	cap5	五	-	-	P44	-	
NZ5	raw milk	cap8	十	gyrA-gyrB-ermB	hld-cna	P7	t189	
NZ6	raw milk	*	七	gyrA-aphA-tetM	-	P35	-	
NZ7	raw milk	cap8	八	gyrA-tetM-ermC-class I-Tn 5801	hlb-hld-clfA	P24	t6367	
NZ8	raw milk	cap5	八	gyrA-aphA-tetM-class II	-	P35	-	

NZ9	raw milk	cap8	七	gyrA-gyrB-grlA-grlB-aphA-tetM-ermB-rpoB-Tn 5801	hla-hlb-hld-lukED-cna-ebp-clfA-clfB	P19	t164	
NZ10	raw milk	cap8	七	gyrA-gyrB-grlA-grlB-aphA-tetM-ermB-ermC-rpoB- nu(A)	hlb-lukED-cna	P23	t3380	
NZ11	raw milk	cap5	五	gyrA-gyrB-grlA-grlB-aphA-rpoB	seb-sem-sen-hla-hlb-hld-lukED-ebp-clfA-clfB	P33	t164	
NZ12	raw milk	cap8	八	gyrA-gyrB-grlA-grlB-ermC-rpoB-lnu(A)	hlb-lukED-cna-ebp-clfA-clfB	P25	t189	
NZ13	raw milk	*	十一	tetM	-	P18	-	
NZ14	raw milk	cap5	十	-	-	-	-	
NZ15	raw milk	cap8	十	mecA-gyrA-gyrB-grlB-qnrB-qnrS-aphA-tetM-ermB-Tn 5801	-	P9	t164	I
NZ16	raw milk	cap5	九	gyrA-tetM-Tn 5801	-	-	-	
NZ17	raw milk	cap5	十	oqxA	-	P2	-	
NZ18	raw milk	cap5	八	oqxA	-	P5	-	
NZ19	raw milk	cap5	九	oqxA-aphA-tetM-ermB	-	P2	-	
NZ20	raw milk	cap5	六	-	-	-	t164	
NZ21	raw milk	cap5	五	qnrB-aphA	-	P45	-	
NZ22	raw milk	cap5	九	gyrA-grlA-grlB-acc(6')-aph(2″)-tetM-ermB-rpoB	hlb-hld-lukED-clfA	P28	t8139	
NZ23	raw milk	*	八	gyrA-rpoB	-	P28	-	
NZ24	raw milk	cap5	十	gyrA-acc(6')-aph(2″)-aphA-tetM	cna-clfA	P28	t164	
NZ25	raw milk	cap5	九	gyrA-grlB-aphA-tetM-ermB	hlb-hld-can-ebp-clfA-clfB	-	t164	
NZ26	raw milk	cap5	六	gyrA-tetM	hlb-hld-can-ebp-clfA-clfB	-	t164	
NZ27	raw milk	cap8	九	gyrA-grlA-grlB-acc(6')-aph(2″)-rpoB	hlb-hld-lukED-can-ebp-clfA-clfB	P1	t189	
NC1	raw milk	cap5	六	grlB-qnrS-aphA-tetK-ermB-ermC	hld-lukED	P26	t091	
NC2	raw milk	cap5	七	grlA-aphA-ermB-ermC-rpoB	sej-sep-set-lukED-ebp-clfA-clfB	P26	t091	
NC3	raw milk	cap5	八	gyrA-gyrB-grlA-grlB-aphA-tetK-ermB-ermC-rpoB	sej-sep-set-hlb-hlg-lukED-ebp-clfA-clfB	P26	t091	
NC4	raw milk	cap5	六	gyrA-gyrB-grlB-acc(6')-aph(2″)-aphA-tetK-tetM-ermB-ermC	seb-sed-set-hld-lukED-can-ebp-clfB	P21	t091	
NC5	raw milk	cap8	八	gyrA-gyrB-grlA-grlB-aphA-tetK-tetM-ermB-ermC	seb-see-sep-set-hld-lukED-can-ebp-clfA-clfB	P6	t899	
NC6	raw milk	cap5	八	oqxA-oqxB-gyrA-grlA-grlB-aphA-ermB-rpoB	set-hld-hlg-cna	-	t034	
NC7	raw milk	*	四	aphA	sej-cna	-	t3512	
NC8	raw milk	cap5	八	gyrA-gyrB-grlA-grlB-qnrS-tetK-tetM-ermB-ermC-rpoB	hlb-hld	P26	t034	
NC9	raw milk	*	六	qnrS-aphA	hld	P26	-	
NC10	raw milk	cap5	三	gyrA-gyrB-grlA-grlB-acc(6')-aph(2″)-tetK-ermB-ermC-rpoB	sed-sej-sen-sep-set-hla-hlb-hlg-lukED-ebp-clfA-clfB	P26	t034	
NC11	raw milk	cap5	八	gyrA-gyrB-grlA-grlB-aphA-tetK-tetM-ermC-rpoB	hld-lukED-ebp-clfA-clfB	P26	t034	
NC12	raw milk	cap5	八	gyrA-gyrB-grlA-grlB-aphA-tetK-ermC-rpoB	hlb-hld-ebp-clfA-clfB	P26	t034	
NK1	raw milk	cap5	三	gyrA-gyrB-grlA-grlB-rpoB	seb-see-set-hld-hlg-lukED-can-ebp-clfA-clfB	P8	t189	
NK2	raw milk	cap5	五	gyrA-gyrB-grlA-grlB-aphA-tetM-ermB-rpoB	seb-set-hld-hlg-lukED-can-ebp-clfA-clfB	P8	t189	
NK3	raw milk	cap5	八	qnrS-acc(6')-aph(2″)-aphA-tetM-ermB	seb-sed-sej-sep-set-hlg-lukED-can-ebp-clfB	P8	t8139	
NK4	raw milk	cap5	八	gyrA-gyrB-grlA-grlB-aphA-tetM-ermB-rpoB	seb-set-hld-lukED-can-ebp-clfB	P8	t189	
NK5	raw milk	cap5	九	gyrA-gyrB-grlA-grlB-tetM-ermB-ermC-rpoB	seb-set-hla-hlb-hld-lukED-can-ebp-clfA-clfB	P8	t189	
NK6	raw milk	*	三	gyrA-gyrB-grlA-grlB-aphA-ermB-rpoB	seb-hld	P8	-	
NK7	raw milk	cap5	六	gyrA-gyrB-grlA-grlB-aphA-tetM-ermB-rpoB	seb-see-set-hld-lukED-can-ebp-clfA-clfB	P8	t189	
NK8	raw milk	cap5	五	qnrS-acc(6')-aph(2″)-aphA-tetM-ermB	sed-sej-sep-set-hlg-cna	P8	t6367	
NK9	raw milk	cap5	八	blaZ-gyrA-gyrB-grlA-grlB-acc(6')-aph(2″)-aphA-tetM-ermB-ermC-rpoB-Tn 552	seb-hla-hlb-hld-lukED-can-ebp-clfA-clfB	P8	t189	
NK10	raw milk	cap5	九	gyrA-gyrB-grlA-grlB-rpoB	seb-hld-lukED-can-ebp-clfA-clfB	P8	t189	

NK11	raw milk	cap5	六	gyrA-gyrB-grlA-grlB-tetM-rpoB-Tn 5801	seb-hla-hld-lukED-can-ebp-clfA-clfB	P8	t189
Z1	pig	cap5	八	gyrA-gyrB-grlA-grlB-fexA-acc(6')-aph(2″)-aphA-tetM-ermA-ermB-ermC-rpoB-bcrB	sed-sej-sem-sen-set-hla-hld-lukED-ebp-clfA-clfB	P1	t15075
Z2	pig	cap5	八	gyrA-grlA-grlB-fexA-ant(4')-Ia-aphA-tetK-tetM-ermA-ermB-ermC-rpoB-bcrB-lnu(A)-Tn 552	sep-hla-hld-lukED-can-ebp-clfA-clfB	P4	t091
Z3	pig	cap5	八	gyrA-grlA-grlB-fexA-acc(6')-aph(2″)-aphA-ermA-ermB-ermC-rpoB-bcrB-	sed-sej-hla-hld-lukED-ebp-clfA-clfB	P1	t15075
Z4	pig	cap5	三	gyrA-gyrB-grlA-grlB-ermC-rpoB	sed-sej-sem-sen-set-hla-hlb-hld-lukED-ebp-clfA-clfB	P1	t15075
Z5	pig	cap5	八	gyrA-gyrB-grlA-grlB-fexA-acc(6')-aph(2″)-ant(4')-Ia-aphA-tetK-tetM-ermA-ermB-ermC-rpoB-bcrB-lnu(A)-class I	sed-sej-hla-hld-lukED-ebp-clfA-clfB	P4	t15075
Z6	pig	cap5	九	gyrA-gyrB-grlA-grlB-fexA-acc(6')-aph(2″)-ant(4')-Ia-aphA-tetK-tetM-ermA-ermB-ermC-rpoB-bcrB-lnu(A)	sed-sej-hla-hld-ebp-clfA-clfB	P1	t15075
Z7	pig	cap5	七	gyrA-gyrB-grlA-grlB-fexA-ant(4')-Ia-ermC-rpoB-Tn 552	hla-hlb-hld-ebp-clfA-clfB	P32	t899
Z8	pig	cap5	九	gyrA-gyrB-grlA-grlB-fexA-acc(6')-aph(2″)-ant(4')-Ia-aphA-tetK-tetM-ermA-ermB-ermC-rpoB-bcrB-lnu(A)	sed-sej-sep-hla-hld-lukED-can-clfA	P4	t034
Z9	pig	cap5	八	gyrA-grlA-grlB-fexA-acc(6')-aph(2″)-ant(4')-Ia-aphA-tetK-tetM-ermA-ermB-ermC-rpoB-bcrB-lnu(A)-optrA	sep-set-hla-hlb-hld-hlg-lukED-can-ebp-clfA-clfB	P4	t034
Z10	pig	cap5	六	gyrA-gyrB-grlA-grlB-ant(4')-Ia-tetK-ermC-rpoB-lnu(A)-Tn 552	sep-set-hla-hld-can-ebp-clfA-clfB	P35	t091
Z11	pig	cap5	十	gyrA-gyrB-grlA-grlB-fexA-acc(6')-aph(2″)-ant(4')-Ia-aphA-tetK-tetM-ermA-ermB-ermC-rpoB-bcrB-lnu(A)-optrA-Tn 552	sed-sej-sep-set-hla-hlb-hld-hlg-lukED-can-ebp-clfA-clfB	P5	t034
Z12	pig	cap5	九	gyrA-gyrB-grlA-grlB-fexA-ant(4')-Ia-ermC-rpoB-Tn 552	hla-hlb-hld-ebp-clfA-clfB	P5	t3512
Z13	pig	cap8	七	gyrA-gyrB-grlA-grlB-ant(4')-Ia-tetK-ermC-rpoB-lnu(A)-Tn 552	sep-hla-ebp-clfA-clfB	P26	t091
Z14	pig	cap5	六	gyrA-gyrB-grlA-grlB-rpoB	hla-clfA	P27	t034
Z15	pig	cap5	八	gyrA-gyrB-grlA-grlB-fexA-acc(6')-aph(2″)-aphA-tetM-ermA-ermB-rpoB-bcrB-class I	sed-see-sej-set-hla-hlb-hld-hlg-lukED-can-ebp-clfA-clfB	-	t034
Z16	pig	cap5	十	gyrA-gyrB-grlA-grlB-fexA-acc(6')-aph(2″)-aphA-tetK-etM-ermA-ermB-ermC-rpoB-bcrB-optrA-class I	sed-sej-sem-sen-sep-set-hla-hlb-hld-lukED-can-ebp-clfA-clfB	P2	t15075
Z17	pig	cap8	七	gyrA-gyrB-grlA-grlB-ant(4')-Ia-tetK-ermC-rpoB-lnu(A)	sep-hla-hld-lukED-ebp-clfA-clfB	P5	t091
Z18	pig	cap5	九	gyrA-gyrB-grlA-grlB-fexA-acc(6')-aph(2″)-aphA-tetK-tetM-ermA-ermB-ermC-rpoB-bcrB	sed-sej-hla-hld-lukED-ebp-clfA-clfB	P1	t15075
Z19	pig	cap5	十	gyrA-gyrB-grlA-grlB-fexA-acc(6')-aph(2″)-aphA-tetM-ermA-ermB-ermC-rpoB-bcrB	hld-lukED-ebp-clfA-clfB	P2	t15075
Z20	pig	cap5	七	gyrA-gyrB-grlA-grlB-ermC-rpoB	sed-sej-hla-hld-lukED-ebp-clfA-clfB	P2	t15075
Z21	pig	cap8	七	gyrA-gyrB-grlA-grlB-ant(4')-Ia-tetK-ermC-rpoB-lnu(A)-Tn 552	sep-hla-hld-ebp-clfA-clfB	P13	t091
Z22	pig	cap5	十一	gyrA-gyrB-grlA-grlB-fexA-aac(6’)-Ib-cr-acc(6')-aph(2″)-aphA-tetM-ermA-ermB-ermC-bcrB-class I	sed-sej-hld-lukED-ebp-clfA-clfB	P45	t15075
J1	chicken	cap5	十	blaZ-gyrA-gyrB-grlA-grlB-fexA-acc(6')-aph(2″)-ant(4')-Ia-aphA-tetK-tetM-ermA-ermB-ermC-rpoB-bcrB-optrA	sep-set-hla-hlb-hld-hlg-lukED-can-ebp-clfA-clfB	P12	t034
J2	chicken	cap5	四	gyrA-gyrB-grlA-grlB-ermC-rpoB	sed-sej-hla-hld-lukED-ebp-clfA-clfB	P1	t2646
J3	chicken	cap5	十	gyrA-gyrB-grlA-grlB-fexA-acc(6')-aph(2″)-aphA-tetM-ermB-rpoB-bcrB-optrA	sed-sej-sen-hla-hld-lukED-ebp-clfA-clfB	P1	t15075
J4	chicken	cap5	八	grlA-grlB- acc(6')-aph(2″)-ermB-rpoB-lnu(A)	set-hla-hlb-hld-hlg-can-ebp-clfA-clfB	-	t034
J5	chicken	cap5	十	blaZ-gyrA-gyrB-grlA-grlB-fexA-acc(6')-aph(2″)-aphA-tetM-ermA-ermB-ermC-rpoB-bcrB	sed-sej-sem-sen-hla-hld-lukED-ebp-clfA-clfB	P1	t15075
J6	chicken	cap5	九	blaZ-gyrA-gyrB-grlA-grlB-fexA-acc(6')-aph(2″)-aphA-tetM-ermA-ermB-ermC-rpoB-bcrB-optrA	sed-sej-sem-sep-set-hla-hlb-hld-lukED-ebp-clfB	P1	t15075

J7	chicken	cap5	五	blaZ-gyrA-gyrB-grlA-grlB-ermC-rpoB	sed-sej-set-hla-hlb-hld-lukED-ebp-clfA-clfB	P1	t15075	
J8	chicken	cap5	十	gyrA-gyrB-grlA-grlB-fexA-acc(6')-aph(2″)-aphA-tetM-ermA-ermB-ermC-rpoB-bcrB	sed-sej-hla-lukED-ebp-clfB	P1	t15075	
J9	chicken	cap5	四	gyrA-gyrB-grlA-grlB-ermC-rpoB	sed-sej-sem-sen-set-hla-hld-lukED-ebp-clfA-clfB	P1	t15075	
J10	chicken	cap8	十	blaZ-gyrA-gyrB-grlA-grlB-ant(4')-Ia-tetK-ermC-rpoB	sep-hla-hld-ebp-clfA-clfB	P4	t034	
J11	chicken	cap5	十	gyrA-gyrB-grlA-ant(4')-Ia-tetK-ermC-rpoB-lnu(A)	sed-sej-sem-sen-sep-hla-hld-lukED-ebp-clfB	P1	t15075	
J12	chicken	cap5	十	blaZ-gyrA-gyrB-grlA-grlB-fexA-acc(6')-aph(2″)-ant(4')-Ia-aphA-tetM-ermA-ermB-ermC-rpoB-bcrB	sed-sej-hla-hld-lukED-ebp-clfA-clfB	P1	t15075	
J13	chicken	cap5	四	gyrA-gyrB-grlA-grlB-ermC-rpoB	sed-sej-sem-sen-set-hla-hlb-lukED-ebp-clfA-clfB	P1	t15075	
J14	chicken	*	六	mecA-gyrA-ant(4')-Ia-rpoB	-	P38	-	II
J15	chicken	cap5	十一	oqxA-oqxB-gyrA-gyrB-grlA-grlB-fexA-aac(6')-Ib-cr-acc(6')-aph(2″)-ant(4')-Ia-aphA-tetM-ermA-ermB-ermC-bcrB-optrA-class I	sed-sej-sen-set-hla-hld-lukED-ebp-clfA-clfB	P1	t15075	
1	waterfowl	*	十一	oqxB-qnrA-qnrB-qnrS-acc(6')-aph(2″)-aphA-tetK-tetM-ermA-ermB-vga(A)-lnu(B)-class I-class II-class III-Tn 916-like-Tn 5801	-	P43	t189	
2	waterfowl	*	六	aac(6')-Ib-cr-qnrA-qnrB-acc(6')-aph(2″)-ant(4')-Ia-aphA-tetM-ermA-ermB-vga(A)- class I-class II-class III-Tn 916-like-Tn 5801	-	P41	-	
3	waterfowl	cap8	十	aac(6')-Ib-cr-qnrA-qnrB-qnrS-acc(6')-aph(2″)-ant(4')-Ia-aphA-tetM-ermA-ermB-vga(A)-bcrB-optrA-class I-class II-class III-Tn 916-like-Tn 5801	cna	P39	t189	
4	waterfowl	*	十一	gyrB-aac(6')-Ib-cr-qnrA-acc(6')-aph(2″)-ant(4')-Ia-aphA-tetK-tetM-ermA-ermB-vga(A)-bcrB-optrA-class I-class II-class III-Tn 916-like-Tn 5801	cna	P36	t189	
5	waterfowl	*	十	oqxB-qnrA-qnrB-qnrS-acc(6')-aph(2″)-aphA-tetM-ermA-ermB-vga(A)-optrA-class I-class II-class III-Tn 5801	cna	-	t189	
6	waterfowl	*	十一	oqxB-qnrA-acc(6')-aph(2″)-ant(4')-Ia-aphA-tetK-tetM-ermA-ermB-vga(A)-class I-class II-class III-Tn 916-like-Tn 5801	-	P39	-	
7	waterfowl	*	九	gyrA-qnrA-acc(6')-aph(2″)-ant(4')-Ia-aphA-tetM-ermA-ermB-vga(A)-class I-class II-class III-Tn 5801	-	-	-	
8	waterfowl	cap8	十	oqxB-aac(6')-Ib-cr-qnrA-qnrS-acc(6')-aph(2″)-aphA-tetM-ermA-ermB-vga(A)-vga(C)-class I-class II-class III-Tn 916-like-Tn 5801	see-set	P22	t3512	
9	waterfowl	*	七	oqxB-qnrA-acc(6')-aph(2″)-aphA-tetM-ermA-ermB-vga(A)-vga(C)-lnu(B)-class I-class II-class III-Tn 916-like-Tn 5801	see-set	-	t3512	
10	waterfowl	*	十一	oqxB-aac(6')-Ib-cr-qnrA-acc(6')-aph(2″)-ant(4')-Ia-aphA-tetM-ermA-ermB-vga(A)-vga(C)-lnu(B)-optrA-class I-class II-class III	sed-see-set-cna	P40	t189	
11	waterfowl	*	十	oqxB-gyrA-qnrA-acc(6')-aph(2″)-aphA-tetM-ermB-vga(C)-class I-class II-class III-Tn 916-like	set	P42	t3512	
12	waterfowl	cap5	十一	oqxB-gyrB-aac(6')-Ib-cr-qnrA-acc(6')-aph(2″)-aphA-tetM-ermA-ermB-ermC-vga(A)-lnu(B)-class I-class II-class III	-	P3	t2224	
13	waterfowl	cap5	十	oqxB-qnrA-acc(6')-aph(2″)-aphA-tetM-ermA-ermB-vga(A)-class I-class II-class III-Tn 916-like-Tn 5801	lukED-ebp-clfA-clfB	P15	t267	
ZY1	human	cap8	八	mecA-gyrA-grlA-grlB-aphA-ermB-rpoB-class I	seb-set-hla-hlb-hld-pvl-ebp-clfA-clfB	P11	t437	IVa
ZY2	human	cap5	十一	gyrA-grlA-aphA-ermA-rpoB-class I-Tn 5801	sea-sec-set-tsst-hld-lukED-can-ebp-clfA-clfB	P14	t030	
ZY3	human	cap5	十一	mecA-gyrA-grlA-grlB-tetM-ermA-rpoB-class I-Tn 5801	sea-sec-tsst-hld-lukED-can-ebp-clfA-clfB	P14	t030	IVa
ZY4	human	cap5	十	rpoB-Tn 5801	sea-sec-sen-set-tsst-hla-hlb-hld-lukED-can-ebp-clfA-clfB	P17	t030	
ZY5	human	*	五	acc(6')-aph(2″)-tetK-tetM-ermB-rpoB-Tn 916-like	-	P16	-	

ZY6	human	cap8	七	gyrA-gyrB-grlA-grlB-ermC-rpoB-class I	sep-set-hla-hlb-hld-lukED-pvl-ebp-clfA-clfB	P14	t3155	
ZY7	human	cap8	九	mecA-gyrA-gyrB-grlA-grlB-tetK-ermB-ermC-rpoB-class I-class II	seb-sep-hla-hld-lukED-pvl-ebp-clfA-clfB	P16	t3155	IVa
ZY8	human	*	五	-	-	P37	-	
ZY9	human	cap8	三	rpoB	hla-pvl-clfA	P16	t3155	
ZY10	human	cap8	九	mecA-gyrA-grlA-grlB-ermC-rpoB-class II	seb-hla-hlb-hld-lukED-pvl-ebp-clfA-clfB	P16	t3155	III
ZY11	human	*	十	gyrA-gyrB-grlA-grlB-tetK-ermC-rpoB-class II	clfA	P34	t2193	
ZY12	human	*	六	mecA-gyrA-grlA-grlB-ermC-rpoB-class II	ebp-clfA-clfB	P16	t3155	III
ZY13	human	cap8	七	mecA-gyrA-gyrB-grlA-grlB-aphA-tetK-ermB-rpoB	Hlb-hld-pvl-ebp-clfA-clfB	P10	t3527	-

"*" non-cap5 and non-cap8

"-" unable to typing or not determined
