# Supplementary material for: Prevalence, resistance pattern, and molecular characterization of Staphylococcus aureus isolates from healthy animals and sick populations in Henan Province, China
Source: Gut Pathog. 2018 Jul 17;10:31. doi: 10.1186/s13099-018-0254-9 (PMC6048774; doi:10.1186/s13099-018-0254-9)
Supplement: Supplementary file 2 — Additional file 2: Table S2. A list of the 33 tested antimicrobials, their classes, and the concentrations used for susceptibility testing of S. aureus. [file 13099_2018_254_MOESM2_ESM.pdf]

Table S2. A list of the thirty-three tested antimicrobials, their classes, and the concentrations used for susceptibility testing of *Staphylococcus aureus*

Antimicrobial categories	Antibiotics	Abbreviation	Content (%)	Concentration range (μg/mL)
Penicillins	penicillin	PEN	90%	0.125-256
	methicillin	MEC	85%	0.25-512
	oxacillin	OXA	90%	0.25-512
Cephalosporins	ceftriaxone	CRO	90%	0.0625-128
	ceftiofur	CEF	98%	0.0625-128
	cefquinome	CEQ	90%	0.0625-128
	cefepime	CEP	90%	0.0625-128
β-lactams/β-Lactamase inhibitors	Imipenem	IMP	90%	0.0625-128
	Cefopcrazone and Sulbactam Sodium（2:1）	-	90%	0.0625-128
	Piperacillin and Tazobactam Sodium（4:1）	-	90%	0.0625-128
Fluoroquinolones	ciprofloxacin	CIP	88.4%	0.0625-128
	enrofloxacin	ENO	98%	0.0625-128
	levofloxacin	LEV	90%	0.0625-128
Aminoglycosides	gentamicin	GEN	65%	0.25-512
	amikacin	AMK	60%	0.0625-128
	neomycin	NEM	65%	0.0625-128
Amphenicols	chloramphenicol	CHL	90%	0.0625-128
	florfenicol	FLO	98%	0.0625-128
Macrolides	tylosin	TYL	95%	0.0625-128
	erythromycin	ERY	90%	0.0625-128
Lincosamides	Lincomycin	LIN	90%	0.0625-128
Tetracyclines	tetracycline	TET	87.8%	0.25-512
	terramycine	TER	85%	0.0625-128
	doxycyclin	DOX	80%	0.0625-128
	tigecycline	TGC	94%	0.0625-128
Quinoxalines	olaquindox	OLA	80%	0.0625-128
	mequindox	MEQ	80%	0.0625-128
independent class of antibiotics	fosfomycin	FOS	90%	0.25-512
Rifamycines	rifampicin	RIF	90%	0.0625-128
polypeptides	vancomycin	VAN	90%	0.0625-128
	Bacitracin	BAT	61 units per mg	0.25-512
	antimicrobial peptide	APT	90%	0.0625-128
Oxazolidinones	linezolid	LZD	99%	0.0625-128
