# Supplementary material for: Prevalence, resistance pattern, and molecular characterization of Staphylococcus aureus isolates from healthy animals and sick populations in Henan Province, China
Source: Gut Pathog. 2018 Jul 17;10:31. doi: 10.1186/s13099-018-0254-9 (PMC6048774; doi:10.1186/s13099-018-0254-9)
Supplement: Supplementary file 3 — Additional file 3: Table S3. Oligonucleotide sequences, primers, and targets for polymerase chain reaction amplification of antibiotic resistance genes and integrons in S. aureus isolates. [file 13099_2018_254_MOESM3_ESM.pdf]

Table S3. Oligonucleotide Sequences, primers, and targets for Polymerase Chain Reaction Amplification of Antibiotic resistance genes and integrons in *Staphylococcus aureus* Isolates

Gene	Primer name	Oligonucleotide sequence (5′→3’)	Amplicon size (bp)	Reference
oqxA	oqxA-F	GGGGAAACATTCACCTGAC	407	This study
	oqxA-R	ACGGGAGACGAGGTTGGTA		
oqxB	oqxB-F	TTCTCCCCCGGCGGGAAGTAC	513	This study
	oqxB-R	CTCGGCCATTTTGGCGCGTA		
ermA	ermA-F	GTTCAAGAACAATCAATACAGAG	421	1
	ermA-R	GGATCAGGAAAAGGACATTTTAC		
ermB	ermB-F	GAAAAGGTACTCAACCAAATA	639	1
	ermB-R	AGTAACGGTACTTAAATTGTTTAC		
ermC	ermC-F	GCTAATATTGTTTAAATCGTCAATTCC	572	1
	ermC-R	GGATCAGGAAAAGGACATTTTAC		
blaZ	blaZ-F	TCAAACAGTTCACATGCC	800	This study
	blaZ-R	TTCATTACACTCTGGCG		
vanA	vanA-F	CATGACGTATCGGTAAAATC	885	2
	vanA -R	ACCGGGCAGCGTATTGAC		
fosB	fosB-F	ACCGGTACTTTACAAGAGCGT	660	This study
	fosB-R	AACAGCACCATCACTTCCTT		
rpoB	rpoB-F	CCGTATCGGTTTATCAAGAATG	432	This study
	rpoB-R	TCAACTTTACGATATGGTGTTT		
cfr	cfr-F	TGAAGTATAAAGCAGGTTGGGAGTCA	746	3
	cfr-R	ACCATATAATTGACCACAAGCAGC		
tetK	tetK-F	TAGGGGGAATAATAGCACATT	587	4
	tetK-R	AATCCGCCCATAACAAATA		
tetM	tetM-F	GAACTCGAACAAGAGGAAAGC	717	4
	tetM-R	ATGGAAGCCCAGAAAGGAT		
aac(6’)-Ib-cr	aac(6’)-Ib-cr -F	TTGCGATGCTCTATGAGTGGCTA	482	This study
	aac(6’)-Ib-cr -R	CTCGAATGCCTGGCGTGTTT		
qepA	qepA-F	CTGCAGGTACTGCGTCATG	403	This study
	qepA-R	CGTGTTGCTGGAGTTCTTC		
qnrA	qnrA-F	CAAGAGGATTTCTCACGCCAG	628	This study
	qnrA-R	AATCCGGCAGCACTATTACTCC		
qnrB	qnrB-F	AGCGGCACTGAATTTATCGG	418	This study
	qnrB-R	CGCAATGTGTGAAGTTTGCT		
qnrC	qnrC-F	GGGTT GTACA TTTAT TGAATCG	449	5

	qnrC-R	AATCC ACTTT ACGAG GTTCT		
qnrD	qnrD-F	CACGAGATCAATTTACGGGGAA	572	This study
	qnrD-R	CGCCTGCTCTCCATCCAAC		
qnrS	qnrS-F	CATTGAACAGGGTGATATCGAA	395	This study
	qnrS-R	ATAAATTGGCACCCTGTAGGC		
gyrA	gyrA-F	AATGAACAAGGTATGACACC	203	This study
	gyrA-R	TACGCGCTTCAGTATAACGC		
gyrB	gyrB-F	CAGCGTTAGATGTAGCAAGC	231	This study
	gyrB-R	CCGATTCCTGTACCAAATGC		
grlA	grlA-F	ACTTGAAGATGTTTTAGGTGAT	538	This study
	grlA-R	TTAGGAAATCTTGATGGCAA		
grlB	grlB-F	CGATTAAAGCACAACAAGCAAG	353	This study
	grlB-R	CATCAGTCATAATAATTACTC		
acc(6')-aph(2″)	acc(6')-aph(2″)-F	CCAAGAGCAATAAGGGCATACC	675	6
	acc(6')-aph(2″)-R	ACCCTCAAAAACTGTTGTTGC		
ant(4')-Ia	ant(4')-Ia-F	GGAAGCAGAGTTCAGCCATG	266	6
	ant(4')-Ia-R	TGCCTGCATATTCAAACAGC		
aphA	aphA-F	ACAGCCGGTATAAAGGGACCACC	382	7
	aphA-R	AAAATCATACAGCTCGCGCGGATC		
vga(A)	vga(A)-F	CTTCAATTGGGATCCTCAGGATAGG	631	8
	vga(A)-R	GTTATGGTACCTTCTTGTTAGG		
vga(B)	vga(B)-F	GAATAAGGCGCAAGGAATGA	601	9
	vga(B)-R	TAGCTTGGCAAAAGCAACCT		
vga(C)	vga(C)-F	TAAGTTCATCGGAAGCAA	671	9
	vga(C)-R	GGATTCAAACGCCTCTAT		
vga(D)	vga(D)-F	CAACTGGAGCGAGCTGTTA	201	10
	vga(D)-R	GACAGCCGGATAATCTTTTG		
lnu(A)	lnu(A)-F	GGTGGCTGGGGGGTAGATGTATTAACTGG	-	11
	lnu(A)-R	GCTTCTTTTGAAATACATGGTATTTTTCGATC		
lnu(B)	lnu(B)-F	CCTACCTATTGTTTGTGGAA	-	11
	lnu(B)-R	ATAACGTTACTCTCCTATTC		
lnu(C)	lnu(C)-F	ACTGTCGCAGAGCAGGAAAGCC	-	11
	lnu(C)-R	AGCATCTACACCCCAGCCACCA		
lnu(D)	lnu(D)-F	ACGGAGGGATCACATGGTAAATAAAGC	-	11
	lnu(D)-R	CCTGTCTTTATCGTCCTTCCAAACCGT		
bcrA	bcrA-F	CCGCAATGAAAATGATGTTG	584	12

	bcrA-R	TGCGGCTATCTTACCATCTG		
bcrB	bcrB-F	AAAGAAACCGACTGCTGATA	489	12
	bcrB-R	GCTTACTTGTATAGCAGAGA		
bcrD	bcrD-F	GCGAAGCGTTTAAGGAAATG	482	12
	bcrD-R	TGGCACAGCAAGAAAGAATG		
bcrR	bcrR-F	TAACGCAGGAACAACTTGC	461	12
	bcrR-R	CAAAGCGGTAATGGTGAGG		
class I	class I-F	CCTCCCGCACGATGATC	288	13
	class I-R	TCCACGCATCGTCAGGC		
class II	class II-F	GTAGCAAACGAGTGACGAAATG	788	13
	class II-R	CACGGATATGCGACAAAAAGGT		
class III	class III-F	GCCTCCGGCAGCGACTTTCAG	979	13
	class III-R	ACGGATCTGCCAAACCTGACT		
Tn 552	Tn 552-F	CTAACTAATTTTTCTGAAGCCAAACG	1360	7
	Tn 552-R	TTTAAGTGTTTTCTTCTCTGACTACG		
Tn 5801	Tn 5801-F	CCGATATTGAGCCTATTGATGTG	722	14
	Tn 5801-R	GTCCATACGTTCCTAAAGTCGTC		
Tn 916-like	Tn 916-like-F	GCCATGACCTATCTTATA	1057	14
	Tn 916-like-R	CTAGATTGCGTCCAA		
optrA	optrA-F	GCACCAGACCAATACGATACAA	794	This study
	optrA-R	TCCTTCTTAACCTTCTCCTTCTCA		
fexA	fexA-F	TTGGGAAGAATGGTTCAGGG	977	This study
	fexA-R	ATCGGCTCAGTAGCATCACG		

References

1. Lim JA, Kwon AR, Kim SK, Chong Y, Lee K, et al. Prevalence of resistance to macrolide, lincosamide and streptogramin antibiotics in Gram-positive cocci isolated in a Korean hospital. J Antimicrob Chemother. 2002;49(3):489-95.

2. Patel R, Uhl JR, Kohner P, Hopkins MK, Rd FRC. Multiplex PCR detection of vanA, vanB, vanC-1, and vanC-2/3 genes in *enterococci*. J Clin Microbiol.1997;35(3):703-7.

3. Kehrenberg C, Schwarz S. Distribution of florfenicol resistance genes *fexA* and *cfr* among chloramphenicol-resistant *Staphylococcus* isolates. Antimicrob Agents Chemother. 2006;50(4):1156-63.

4. Weigel LM, Donlan RM, Shin DH, Jensen B, Clark NC, et al. High-level vancomycin-resistant *Staphylococcus aureus* isolates associated with a polymicrobial biofilm. Antimicrob Agents Chemother. 2007;51(1):231-8.

5. Wang MH, Guo QL, Xu XG, Wang XY, Ye XY, et al. New plasmid-mediated quinolone resistance gene, *qnrC*, found in a

clinical isolate of *Proteus mirabilis*. Antimicrob Agents Chemother. 2009;53(5):1892-7.

6. Kobayashi N, Alam M, Nishimoto Y, Urasawa S, Uehara N, et al. Distribution of aminoglycoside resistance genes in recent clinical isolates of *Enterococcus faecalis*, *Enterococcus faecium* and *Enterococcus avium*. Epidemiol and Infect. 2001; 126(2):197-204.

7. Argudín MA, Mendoza MC, Gonzálezhevia MA, Bances M, Guerra B, et al. Genotypes, Exotoxin Gene Content, and Antimicrobial Resistance of *Staphylococcus aureus* Strains Recovered from Foods and Food Handlers. Appl Environ Microbiol. 2012;78(78): 2930-5.

8. Haroche J, Allignet J, Buchrieser C, El SN. Characterization of a variant of *vga (A)* conferring resistance to streptogramin A and related compounds. Antimicrob Agents Chemother. 2000;44(9):2271-5.

9. Feßler AT, Kadlec K, Hassel M, Hauschild T, Eidam C, et al. Characterization of methicillin-resistant *Staphylococcus aureus* isolates from food and food products of poultry origin in Germany. Appl Environ Microb. 2011;**77**(20):7151-7.

10. Jung YH, Shin ES, Kim O, Yoo JS, Lee KM, et al. Characterization of two newly identified genes, *vgaD* and *vatG*, conferring resistance to streptogramin A in *Enterococcus faecium*. Antimicrob Agents Chemother. 2010;54(11):4744-9.

11. Lozano C, Aspiroz C, Sáenz Y, Ruizgarcía M, Royogarcía G, et al. Genetic environment and location of the *lnu (A)* and *lnu (B)* genes in methicillin-resistant *Staphylococcus aureus* and other *staphylococci* of animal and human origin. J Antimicrobl Chemother. 2012;67(12):2804-8.

12. Matos R, Pinto VV, Ruivo M, Lopes, MDFS. Study on the dissemination of the *bcrABDR* cluster in *Enterococcus* spp. reveals that the *BcrAB* transporter is sufficient to confer high-level bacitracin resistance. Int J Antimicrob Agents. 2009; 34(2):142-7.

13. Ren C, Zhao Y, Shen Y. Analysis of the effect of integrons on drug-resistant *Staphylococcus aureus* by multiplex PCR detection. Mol Med Rep. 2013;7(3):719-24.

14. De Vries LE, Christensen H, Skov RL, Aarestrup FM, Agersø Y. Diversity of the tetracycline resistance gene *tet (M)* and identification of *Tn916*-and *Tn5801*-like (*Tn6014*) transposons in *Staphylococcus aureus* from humans and animals. J Antimicrob Chemother. 2000;64(3):490-500.
