# Supplementary material for: Prevalence, resistance pattern, and molecular characterization of Staphylococcus aureus isolates from healthy animals and sick populations in Henan Province, China
Source: Gut Pathog. 2018 Jul 17;10:31. doi: 10.1186/s13099-018-0254-9 (PMC6048774; doi:10.1186/s13099-018-0254-9)
Supplement: Supplementary file 4 — Additional file 4: Table S4. Oligonucleotide sequences, primers, and targets for polymerase chain reaction amplification of virulence genes in S. aureus isolates. [file 13099_2018_254_MOESM4_ESM.pdf]

Table S4. Oligonucleotide Sequences, primers, and targets for Polymerase Chain Reaction Amplification of Virulence genes in *Staphylococcus aureus* Isolates

Gene	Primer name	Oligonucleotide sequence(5′→3’)	Amplicon size(bp)	Reference
sea	sea-F	AAAGATTTGCGAAAAAAGTGTGAATT	669	1
	sea-R	TAAATCGTAATTAACCGAAGGTTCTG		
seb	seb-F	TATGATGATAATCATGTATCAGCAA	424	1
	seb-R	CCAAATAGTGACGAGTTAGGTAA		
sec	sec-F	TATGATGATAATCATGTATCAGCAA	315	1
	sec-R	TAAGTACATTTTGTAAGTTCCCATT		
sed	sed-F	CTAGTTTGGTAATATCTCCTTTAAACG	319	1
	sed-R	TTAATGCTATATCTTATAGGGTAAACATC		
see	see-F	AAAGATTTGCGAAAAAAGTGTGAATT	512	1
	see-R	TTGCACCTTACCGCCAAAGCTG		
sej	sej-F	CTCCCTGACGTTAACACTACTAATAA	641	1
	sej-R	TTGTCTGGATATTGACCTATAACATT		
sem	sem-F	ATGCTGTAGATGTATATGGTCTAAG	473	1
	sem-R	CGTCCTTATAAGATATTTCTACATC		
sen	sen-F	ATGAGATTGTTCTACATAGCTGCAAT	680	2
	sen-R	AACTCTGCTCCCACTGAAC		
sep	sep-F	TTAGACAAACCTATTATCATAATGG	272	1
	sep-R	TATTATCATGTAACGTTACACCGCC		
set	set-F	GAAGGTCTACAAGGCCAAAATGTCT	363	3
	set-R	TCAACACATCGCCCATGCGCTCGA		
hla	hla-F	CTGATTACTATCCAAGAAATTCGATTG	209	2
	hla-R	CTTTCCAGCCTACTTTTTTATCAGT		
hlb	hlb-F	GTGCACTTACTGACAATAGTGC	309	2
	hlb-R	GTTGATGAGTAGCTACCTTCAGT		
hld	hld-F	AAGAATTTTTATCTTAATTAAGGAAGGAGTG	111	2
	hld-R	TTAGTGAATTTGTTCACTGTGTCGA		
hlg	hlg-F	GTCATAGAGTCCATAATGCATTTAA	535	2
	hlg-R	CACCAAATGTATAGCCTAAAGTG		
lukED	lukED-F	TGAAAAAGGTTCAAAGTTGATACGAG	269	2
	lukED-R	TGTATTCGATAGCAAAAGCAGTGCA		
pvl	pvl-F	ATCATTAGGTAAAATGTCTGGACATGATCCA	433	4
	pvl-R	GCATCAAGTGTATTGGATAGCAAAAGC		
cna	cna-F	CGGGAGATATGCTACCAGAAGATA	278	3

	cna-R	ATAGCCTTGTGGAATTGTTACATCA		
ebp	ebp-F	AGACCAATCAGAATTAGAACATCA	378	3
	ebp-R	TCAGAAACTGTTGAATGCTCAGTGT		
clfA	clfA-F	AAAACACGCAATTCGGAAAA	855	5
	clfA-R	GCAGTTGAAGTTACACCATTTAAGT		
clfB	clfB-F	TGTCGAATAAGCAGAATAAG	505	5
	clfB-R	GGTGATGATTGTGGTAAATC		
tsst	tsst-F	AGCATCTACAAACGATAATATAAAGG	481	1
	tsst-R	CATTGTTATTTTCCAATAACCACCCG		
eta	eta-F	CTATTTACTGTAGGAGCTAG	741	1
	eta-R	ATTTATTTGATGCTCTCTAT		
etb	etb-F	ATACACACATTACGGATAAT	629	1
	etb-R	CAAAGTGTCTCCAAAAGTAT		
etd	etd-F	AACTATCATGTATCAAGG	376	1
	etd-R	CAGAATTTCCCGACTCAG		

References

1. Argudín MA, Mendoza MC, Gonzálezhevia MA, Bances M, Guerra B, et al. Genotypes, Exotoxin Gene Content, and Antimicrobial Resistance of *Staphylococcus aureus* Strains Recovered from Foods and Food Handlers. Appl Environ Microbiol. 2012;78(78): 2930-5.
2. Jarraud S, Mougel C, Thioulouse J, Lina G, Meugnier H, et al. Relationships between *Staphylococcus aureus* genetic background, virulence factors, *agr* groups (alleles), and human disease. Infect Immun. 2002;70(2):631-41.
3. Babamoussa L, Sina H, Scheftel JM, Moreau B, Saintemarie D, et al. *Staphylococcal* Panton-Valentine leucocidin as a major virulence factor associated to furuncles. PLoS One. 2011;6(10):e25716.
4. Lina G, Piémont Y, Godail-Gamot F, Bes M, Peter MO, et al. Involvement of Panton-Valentine leukocidin-producing *Staphylococcus aureus* in primary skin infections and pneumonia. Clin Infect Dis. 1999;29(5):1128-32.
5. Ote I, Taminiau B, Duprez JN, Dizier I, Mainil JG. Genotypic characterization by polymerase chain reaction of *Staphylococcus aureus* isolates associated with bovine mastitis. Vet Microbiol. 2011;153(3-4):285-292.
